# Donor-Acceptor Dyads and Triads Employing Core-Substituted Naphthalene Diimides: A Synthetic and Spectro (Electrochemical) Study

**DOI:** 10.3390/molecules27248671

**Published:** 2022-12-08

**Authors:** Samuel Quinn, E. Stephen Davies, Nicholas Pearce, Callum Rosenberg, Constance R. Pfeiffer, Georgia R. F. Orton, Neil R. Champness

**Affiliations:** 1 School of Chemistry, University of Nottingham, University Park, Nottingham NG7 2RD, UK; 2 School of Chemistry, University of Birmingham, Edgbaston, Birmingham B15 2TT, UK

**Keywords:** naphthalene diimide, phenothiazine, phenoxazine, electrochemistry, spectroelectrochemistry

## Abstract

Donor-acceptor dyads and triads comprising core-substituted naphthalene diimide (NDI) chromophores and either phenothiazine or phenoxazine donors are described. Synthesis combined with electrochemical and spectroelectrochemical investigations facilitates characterisation of the various redox states of these molecules, confirming the ability to combine arrays of electron donating and accepting moieties into single species that retain the redox properties of these individual moieties.

## 1. Introduction

To further develop electronically or photochemically active organic molecules, it is important to enhance our understanding of synthetic methodologies and to pursue a detailed appreciation of the properties of molecular targets. We have been exploring how it is possible to combine both donor and acceptor groups within a single molecule, with the aim of inferring the properties of both donor and acceptor on a single molecular species. In particular, we are interested to investigate whether the properties of donor and acceptor influence each other, or if the components act independently. With this aim in mind, we have been studying the class of acceptor molecules based on naphthalenetetracarboxylic diimides (NDIs) and their analogues [1,2,3,4].

NDIs are a class of dye molecules that present many opportunities for incorporation into more complex structures [5], leading to their use in various applications including organic electronics [6,7,8] and photovoltaic devices [9,10,11,12,13,14]. In the context of supramolecular chemistry, NDIs have been employed for anion recognition [15,16,17], the creation of metal-organic supramolecular structures [18], metal–organic frameworks [19] and hydrogen-bonded arrays [20,21]. The wide applicability of this class of molecules arises from the combination of the robust NDI core and the potential for chemical modification, coupled with interesting and yet tuneable electrochemical and photochemical properties.

**Scheme 1:**
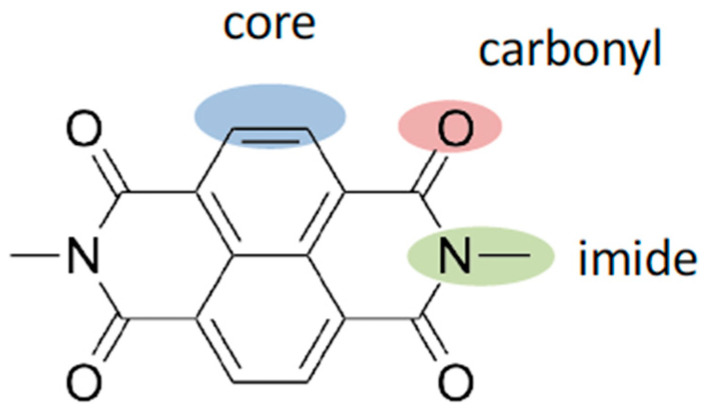
Potential sites for functionalisation of NDI species allowing modification of electrochemical and optical properties.

NDIs can be modified in three main positions: through functionalisation of the imide nitrogen [1], thionation of the carbonyl groups [1] or introduction of substituents directly onto the naphthyl core (Scheme 1) [2,22,23]. Whereas imide functionalisation typically has little effect on the properties of the NDI, due to a node in the molecular orbitals at the imide nitrogen [1], both carbonyl thionation and direct naphthyl core functionalisation can lead to significant modification of the electrochemical and optical properties of the NDI species. Herein, we use naphthyl core substitution to combine NDIs, which can behave as acceptor molecules, with the electron rich organic donor species phenothiazine and phenoxazine. This is achieved through coupling of the donors through a phenylene bridge onto the naphthyl core, by the use of secondary amine groups, which are known to influence the properties of the NDI by conjugating to the aromatic core [2,22,23]. Although some NDI–phenothiazine dyads have been reported [1], little research has been described employing the analogous donor species phenoxazine. Recent studies of the related species naphthalimide–phenothiazine [24,25] or naphthalimide–phenoxazine [26] show promise for creating donor–acceptor dyads with these and related components. Herein we report the synthesis of a series of NDI-based electron donor–acceptor systems composed of an NDI acceptor furnished with phenothiazine or phenoxazine donor appendages in combination with other core-substituents. The electronic and optical properties of these species were probed using cyclic voltammetry and spectroelectrochemistry, establishing the degree of interaction between the NDI manifold and the redox active groups of the appendages. Our approach illustrates the potential to tune the resulting electronic and optical properties using a building-block approach.

## 2. Results and Discussion

### 2.1. Synthesis of Dyads and Triads

The target molecules were synthesised as described below, characterised by a combination of standard spectroscopic techniques and, in some instances, by single crystal X-ray diffraction. All compounds were then probed by electrochemical and spectroelectrochemical techniques allowing the influence of individual components on the molecular properties to be studied.

An amine functionalised phenothiazine species, 10-(4-aminophenyl)-10H-phenothiazine (PTZ-NH_2_), was initially prepared by the literature methods using a Buchwald–Hartwig type reaction to functionalise the phenothiazine with a nitrophenyl group [27]. A slight variation to this approach involved reducing this corresponding nitro-functionalised species to the desired amine using a Pd/C catalyst under an atmosphere of H_2_ at room temperature, rather than the elevated temperatures previously employed. These alternative conditions were found to give a higher yield and allow a simpler, faster work-up procedure than the previous reports. The phenoxazine analogue of 10-(4-aminophenyl)-10H-phenothiazine, 10-(4-aminophenyl)-10H-phenoxazine (POZ-NH_2_), was prepared in order to investigate the effect of modifying the redox properties of the donor species. Although a robust synthesis has recently been reported [28], we used a slightly different approach to prepare POZ-NH_2_. Our initial attempts to replicate the Buchwald–Hartwig type synthesis of PTZ-NH_2_ using a coupling between phenoxazine and 1-iodo-4-nitrobenzene led to decomposition of the phenoxazine component, presumably as a result of the elevated temperatures of this reaction (90 °C). By switching strategy to a copper catalysed Chan–Lam type coupling, which typically progresses well with secondary amines at room temperature, we were able to introduce a nitrophenyl group to the phenoxazine scaffold. This was achieved by reacting the secondary amine of phenoxazine with 4-nitrophenylboronic acid in the presence of copper (II) acetate and pyridine. Subsequent reduction of the nitrophenyl group using a Pd/C catalyst under an atmosphere of H_2_ afforded POZ-NH_2_ in 70% yield. Both PTZ-NH_2_ and POZ-NH_2_ were found to decompose at room temperature in light and were therefore stored in the dark at −18 °C. Due to the success in synthesising POZ-NH_2_ via the Chan–Lam coupling pathway, this method was later applied to the synthesis of PTZ-NH_2_, achieving an overall yield of 83%, an improvement on the literature reports [27].

**Scheme 2:**
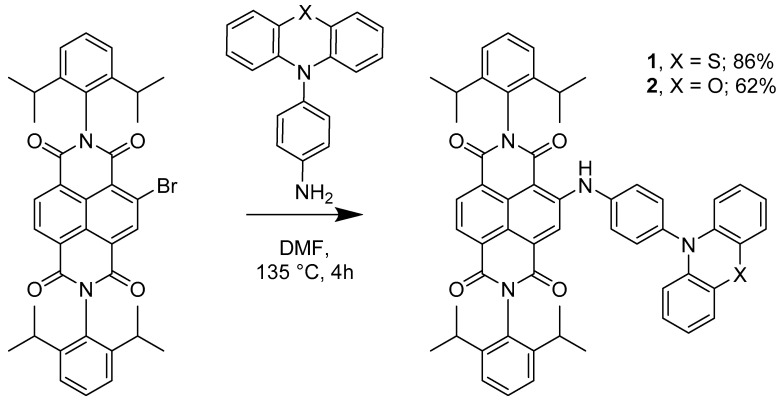
Synthesis of compounds **1** and **2**.

**Figure 1:**
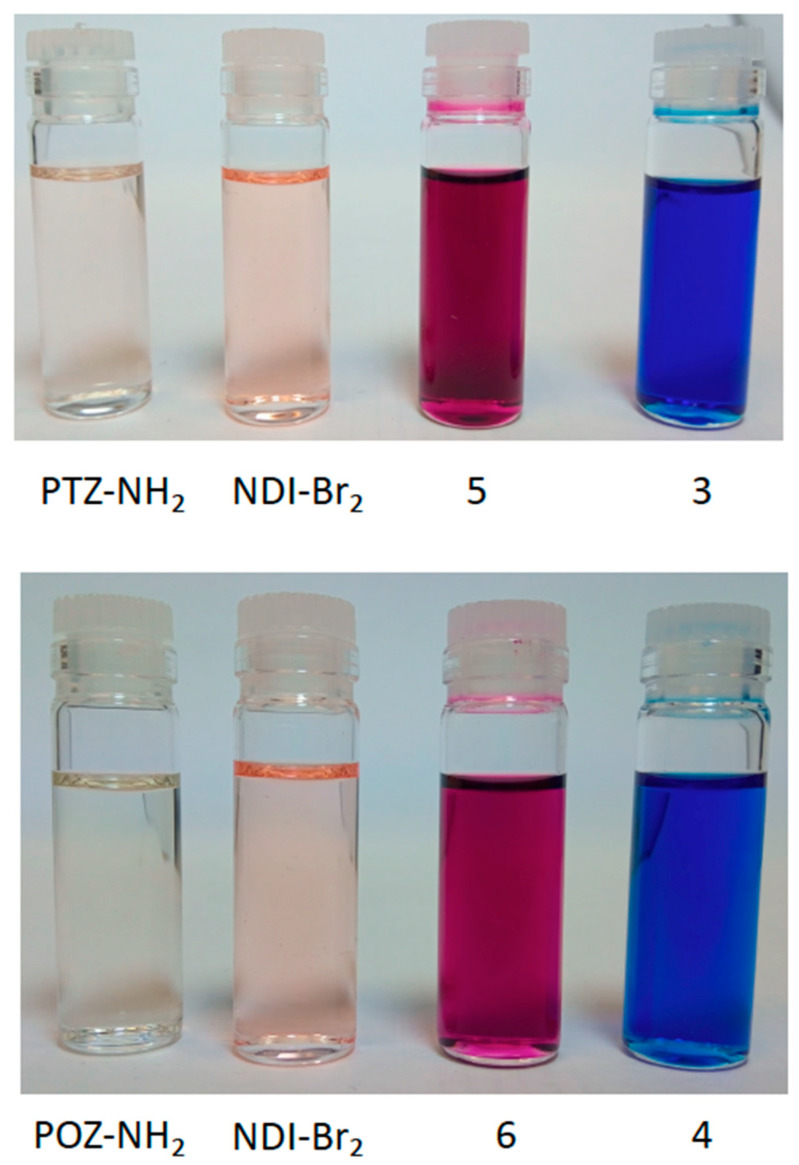
Photographs of CH_2_Cl_2_ solutions of the various compounds. Note the pink colour associated with mono-amino substituted NDIs, **5** and **6**, and di-amino substituted NDIs, **3** and **4**.

**Scheme 3:**
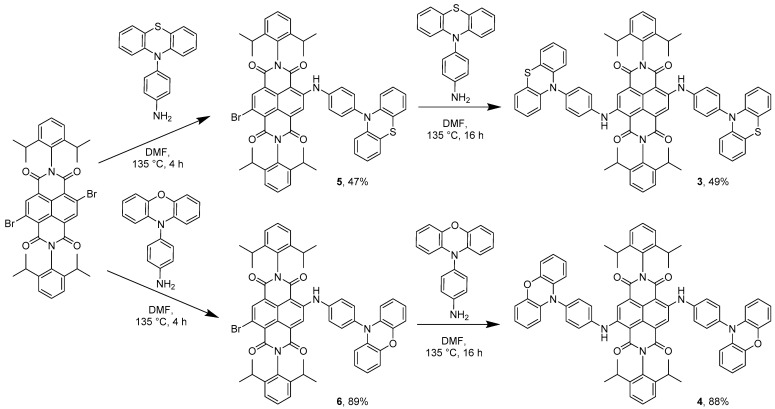
Synthesis of compounds **3–6**.

In order to prepare donor–acceptor dyads involving NDI and either phenothiazine or phenoxazine, we reacted the donor phenothiazine or phenoxazine precursors with either mono-brominated NDI (2-bromo-*N,N’*-bis(2,6-diisopropylphenyl)-1,4,5,8-naphthalenetetracarboxylic diimide) or di-brominated NDI (2,6-dibromo-*N,N’*-bis(2,6-diisopropylphenyl)-1,4,5,8-naphthalenetetracarboxylic diimide) [2,29]. In the case of the mono-substituted systems, reaction of mono-bromo NDI with either PTZ-NH_2_ or POZ-NH_2_ in DMF at 135 °C afforded dyads **1** and **2**, respectively (Scheme 2). Formation of **1** or **2** was accompanied by a colour change from beige to pink, indicating the coupling of the amino group with the naphthyl core of the NDI. Triad **3** was initially prepared by reacting di-brominated NDI with PTZ-NH_2_ (Scheme 3) which gave a mixture of two products: **3** and **5**, corresponding to mono- and di-substitution by the amino-phenothiazine appendage, respectively (see Figure 1 for corresponding colour changes). Indeed, the mono-substituted product **5** was heavily favoured with 47% yield, versus 7% of the di-substituted **3**, thus providing a pathway to asymmetrically substituted NDI triad systems (see below). We reason that the first amine substitution has a deactivating effect on the naphthyl core through increased electron donation, that reduces the nucleophilicity of the second substitution site [2]. Despite this, **3** can be reasonably prepared by further reaction of **5** and PTZ-NH_2_ in DMF at 135 °C. A colour change of the reaction mixture from pink to blue indicated the successful addition of the second amine group to the NDI (see Figure 1).

An analogous reaction was attempted between di-brominated NDI and POZ-NH_2_ under the same reaction conditions as employed for the phenothiazine species that resulted exclusively in the formation of mono-substituted **6**, without any evidence for the formation of di-substituted phenoxazine derivative **4.** Subsequent reaction of **6** with a large excess of POZ-NH_2_ afforded the di-substituted NDI target **4** (Scheme 3). Even when using several equivalents of POZ-NH_2_, the yield of **4** was low due to decomposition of the aminophenoxazine precursor.

**Scheme 4:**
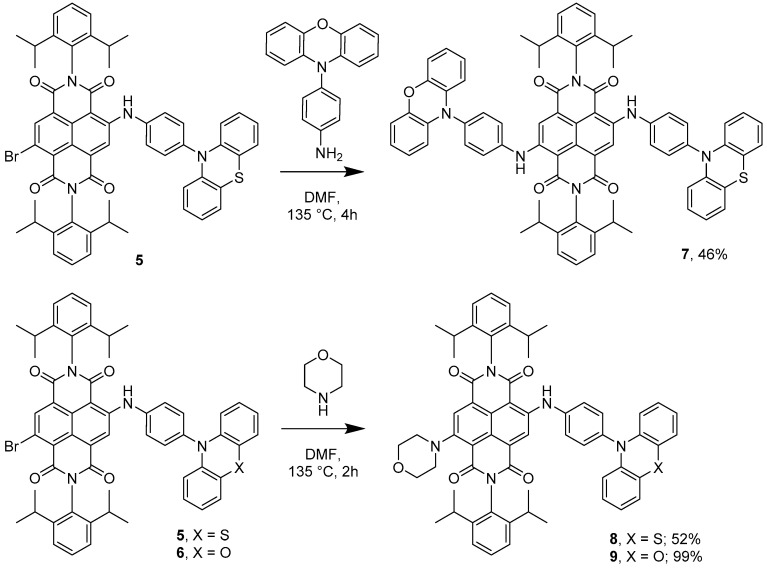
Synthesis of asymmetrically substituted NDI systems. Combining both phenothiazine and phenoxazine appendages (**7**); and morpholine with either phenothiazine (**8**) or phenoxazine (**9**).

Single substitution of di-brominated NDI with either PTZ-NH_2_ or POZ-NH_2_ to give **5** and **6**, respectively, gives rise to compounds with a residual bromo site which can be subsequently replaced with other groups. The effectiveness of this pathway was demonstrated by reacting **5** with POZ-NH_2_ to give a hybrid phenothiazine-NDI-phenoxazine triad **7** (Scheme 3). Additionally, either **5** or **6** can react with morpholine to generate morpholine-NDI-phenothiazine **8** or morpholine-NDI-phenoxazine **9** triads, respectively (Scheme 4). Characterisation of **3**, **4** and **7** by NMR spectroscopy proved to be a challenge due to their poor solubility; suitably concentrated samples could only be achieved by using solvent mixtures containing methylene chloride-*d2* and benzene-*d6*. Toluene-*d8* also proved to be an adequate solvent, although unfortunately led to a significant overlap between the aromatic environments of the sample and the solvent.

**Figure 2:**
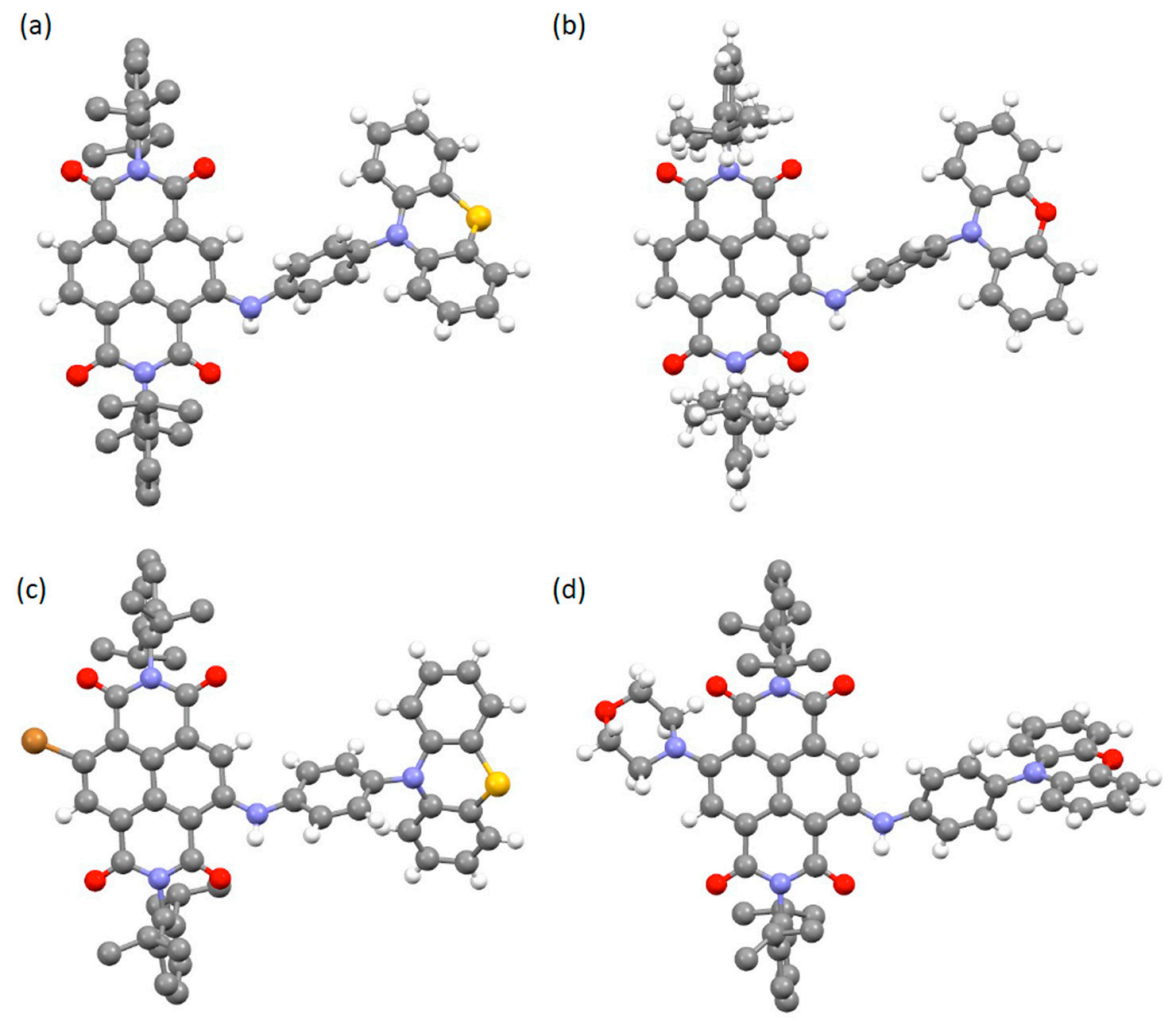
Views of the crystal structures of (**a**) **1**, (**b**) **2**, (**c**) **5** and (**d**) **9**. Note the similarities in the structures but also the variations in the arrangement of the phenothiazine or phenoxazine appendage with respect to the NDI core, illustrating the flexibility of this arm even in the solid-state. In each case the hydrogen atoms of the N-terminal diisopropylphenyl groups are omitted for clarity. C—grey; H—white; O—red; N—blue; S—yellow; Br—brown.

All target compounds were characterised by NMR spectroscopy and mass spectrometry, and in the cases of **1**, **2**, **5** and **9** by single crystal X-ray diffraction (SCXRD). Single crystals were grown by vapour diffusion of either MeOH (**1**, **2**) or hexane (**5**, **9**) into CHCl_3_ solutions of the respective compounds. The single crystal X-ray data confirmed the formula of each compound and allowed for appreciation of the conformations adopted by the dyads (**1**, **2**, **5**) and triad (**9**) (Figure 2). In the solid state, it is readily notable that the conformations of the phenothiazine and phenoxazine groups are different, with the latter being significantly more planar than the former. Thus, the phenoxazine groups in compounds **2** and **9** adopt an angle of 7.79° and 1.94°, respectively, between the two planes formed by the outer phenyl rings of the phenoxazine group. In contrast, angles of 21.7° or 34.76° are observed for the corresponding interplanar geometry of the phenothiazine groups of **1** and **5**, respectively. These angles are consistent with other solid-state structures of phenothiazine [3] or phenoxazine [30,31] derivatives.

The angle adopted between the plane formed by the NDI group and the phenothiazine or phenoxazine appendage is similar for compounds **1**, **2** and **5** with interplanar angles of 24.35°, 23.11° or 21.54°, respectively. This is somewhat surprising considering the flexibility of the amino linker. However, in the solid-state, compound **9** adopts a distinct arrangement with a value of 71.46° for this analogous interplanar angle. It might be anticipated that intermolecular π–π interactions have a significant effect on the conformational arrangement of these molecules, but these are only observed in **5**, with pairs of molecules interacting between the phenyl group of a phenothiazine and the NDI of the adjacent molecule (see Figure S23) and suggesting that π–π interactions are not responsible for affecting the conformation of the molecules. The presence of the morpholino group in **9** may account for the distorted conformation since the morpholino group of one molecule sits in close proximity to the phenoxazine of an adjacent molecule (closest separation of ca. 2.9 Å) and thus close-packing may contribute to the distinct conformation. In all structures, the phenylene bridge that connects the acceptor NDI with the donor phenothiazine or phenoxazine groups is rotated with respect to the planes of each moiety, breaking the conjugation between the acceptor and donor units.

### 2.2. Investigation of Optical and Electrochemical Properties

To investigate the optical and electronic properties of dyads and triads, cyclic voltammetry (CV) and spectroelectrochemical experiments were undertaken. CH_2_Cl_2_ was used as the solvent for these studies due it successfully dissolving all compounds studied at a concentration suitable for cyclic voltammetry, and also providing direct comparison to NDI studies in the literature [3]. Initially, CV measurements were used to establish the redox properties of the various species, see Table 1 for details of redox potentials. For comparison, the cyclic voltammogram of POZ-NH_2_ was recorded, finding that it exhibited a reversible single electron oxidation at 0.25 V (vs Fc^+^/Fc), a small shift from the phenothiazine analogue PTZ-NH_2_ of 0.21 V (vs Fc^+^/Fc) [1].

The donor–acceptor dyads **1** and **2** revealed two one-electron reductions associated with the NDIs in their cyclic voltammograms, occurring at –1.06 V and –1.48 V for **1** and –1.04 V and –1.48 V for **2**. These potentials are similar to those observed for other amine substituted NDIs [1,2,23] indicating that the NDI reduction potentials are primarily affected by the electron donating aniline moiety, and the extended residues do not exert a significant influence. Additionally, both **1** and **2** exhibit a one-electron reversible oxidation based on the phenothiazine (0.33 V vs. Fc^+^/Fc) or phenoxazine (0.36 V vs. Fc^+^/Fc), respectively, and each of these oxidations is shifted to a positive potential relative to PTZ-NH_2_ or POZ-NH_2_.

The cyclic voltammograms of compounds **3**–**9** exhibit the same overall form as those observed for **1** and **2**, with two one-electron reduction steps observed for the NDI component and an oxidation process for the phenothiazine or phenoxazine groups. In the case of **3** and **4** a single oxidation wave is observed, attributed to two overlapping one-electron (non-communicating) oxidations, reflecting the presence of two phenothiazine (**3**) or phenoxazine (**4**) groups. Interestingly, **7**, containing both phenothiazine and phenoxazine groups, gives the same voltammetric profile as **3** and **4**; the individual oxidations based on the phenothiazine and phenoxazine residues are not resolved, and instead a single oxidation process appears, with peak currents at potentials between those of **3** and **4**.

The reduction processes for the hybrid morpholino/phenothiazine (**8**) and morpholino/phenoxazine (**9**) triad systems are observed at more negative potentials relative to **1** and **2**, respectively, as would be expected following the direct attachment to the NDI of the more electron donating morpholino substituent (Table 1) [2]. The oxidation processes in **8** and **9** are shifted to lower potentials than those in **1** and **2**, respectively, but the magnitude of these shifts is small, specifically a shift of 20 mV when comparing **1** and **8** and a shift of 30 mV when comparing **2** and **9**, indicating that further substitutions to the NDI have little effect on the donor properties.

The changes in optical absorption behaviour of dyad and triad molecules following oxidation or reduction were examined using spectroelectrochemical methods. Details of UV/vis absorbance spectra are given in Table S1 and the Supporting Information. The UV/vis spectrum of POZ-NH_2_ following oxidation was also recorded to enable comparison with the dyad/triad species (Figure S28). As the oxidation progresses, the high energy band at 242 nm is seen to deplete and new lower energy bands form, with two bands within the visible range at 412 nm and 532 nm and a weak band emerging above 700 nm. The overall appearance is similar to that previously reported for electrochemically oxidised PTZ-NH2 [1].

**Figure 3:**
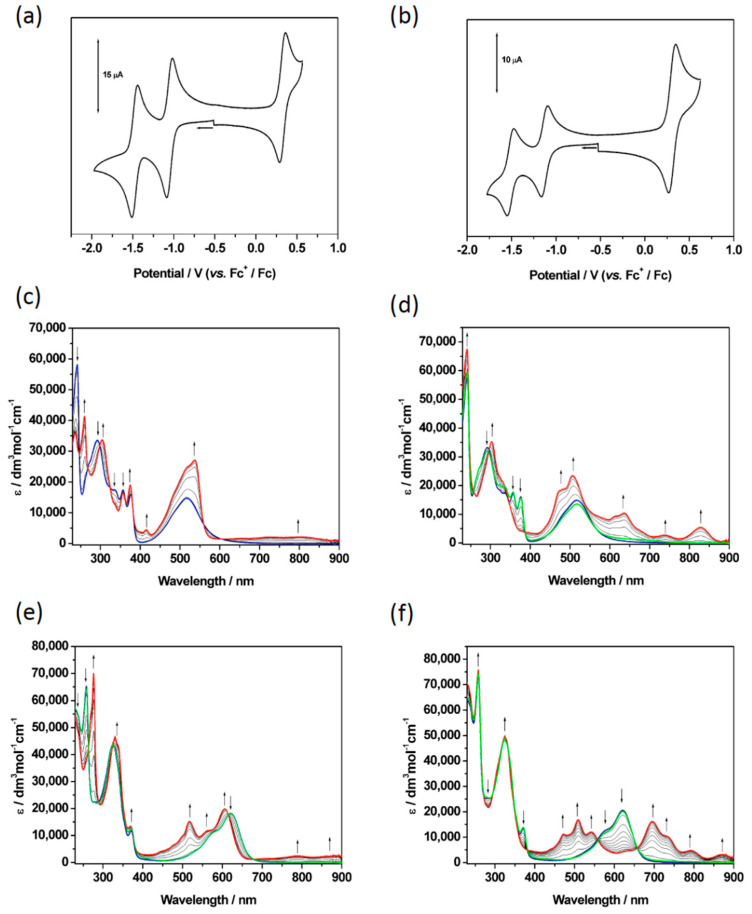
Cyclic voltammograms of (**a**) **1** and (**b**) **3** in CH_2_Cl_2_ with 0.4 M [NBu_4_][BF_4_] as the supporting electrolyte, at a scan rate of 100 mVs^−1^. UV/vis absorption spectra recorded in CH_2_Cl_2_ containing [NBu_4_][BF_4_] (0.4 M) using spectroelectrochemical methods for **2** and **3** at 243 K showing the inter-conversion of (**c**) **2** (blue) to **2^+^** (red) and (**d**) of **2** (blue) to **2^−^** (red) to the regenerated species of **2** (green); (**e**) the inter-conversion of **3** (blue) to **3^2+^** (red) to the regenerated species of **3** (green); (**f**) the inter-conversion of **3** (blue) to **3^−^** (red) to the regenerated species of **3** (green). Arrows show the progress of (**c**,**e**) the oxidation or (**d**,**f**) the reduction.

The oxidation of dyads **1** (Figure S8) and **2** (Figure 3) yielded similar spectra, with a band at high energy depleting and the emergence of new bands at lower energy. Bands within the visible region gain intensity and these are accompanied by the development of a broad absorption above 700 nm and these changes are consistent with the oxidation of the donor species. In reduction, the spectral profiles of dyads **1** (Figure S29) and **2** (Figure 3) are similar. The bands at around 350 nm associated with neutral NDI deplete and the absorption bands characteristic of the monoanionic species, **1^−^** or **2^−^**, at ca. 500 nm, emerge, accompanied by new bands at lower energies. The second reduction of **1** was also probed (Figure S29c) with the resulting dianion, **1^2−^**, sharing similar features to previously reported NDI dianions [2]. The spectra of oxidised and reduced states of **1** and **2** are similar, as might be expected, however, upon regeneration of the parent neutral species, the absorbance bands associated with **2** are slightly less intense, indicating minor chemical decomposition during the redox process. Greater resilience was shown by **1** and the neutral form could be fully regenerated following electrochemical oxidation, indicating that the phenoxazine species, **2**, is less stable than the corresponding phenothiazine species, **1**, under these conditions.

**Figure 4:**
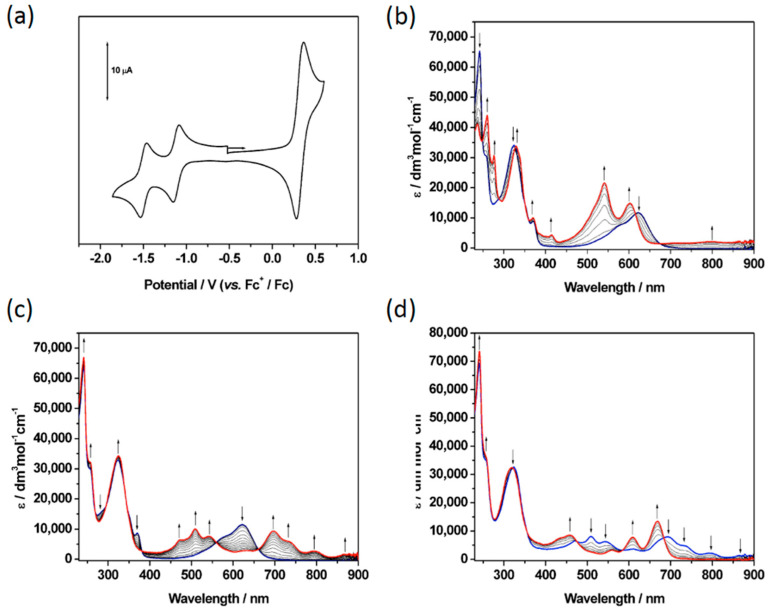
(**a**) Cyclic voltammogram of **7** and (**b**–**d**) UV/vis absorption spectra of **7** recorded in CH_2_Cl_2_ containing [NBu_4_][BF_4_] (0.4 M) using spectroelectrochemical methods at 243 K showing (**b**) the inter-conversion of **7** (blue) to **7^2+^** (red); (**c**) the inter-conversion of **7** (blue) to **7^−^** (red); (**d**) the inter-conversion of **7^−^** (blue) to **7^2−^** (red). Arrows show the progress of (**b**) the oxidation and (**c**/**d**) the reduction.

The oxidations of the triads **3** (Figure 3) and **4** (Figure S30) exhibited similar behaviour and spectral profiles as their dyad analogues, **1** and **2**. Upon regeneration of the neutral form of **3** following oxidation, the intensity of the spectral profile was depleted, indicating that the oxidized species is not chemically stable under these conditions and over the longer timeframe required for spectroelectrochemical study. Although reduction of both **3** (Figure 3f) and **4** (Figure S30b) gave rise to the expected absorption profile, surprisingly, both species proved to be chemically unstable to reduction under these conditions and upon regeneration of the neutral species the corresponding absorption bands showed reduced intensity. This is unexpected behaviour as NDIs typically form very stable anions, indicating that the addition of the second donor species leads to this instability. When examining the hybrid triad system **7** (Figure 4), it was expected that similar changes in optical properties to those in **3** and **4** would be observed upon oxidation and this was confirmed. Interestingly, however, oxidised **7** appears to be more stable than its partner dyads, and the neutral parent species fully regenerated upon reduction. The electrochemically reduced monoanion, **7^−^**, (Figure 4c) displayed similar absorbance behaviour to **3** and **4**, although, as with the oxidation processes, **7** proved to be more chemically stable under these conditions and the neutral species was able to be fully regenerated upon oxidation. Given the stability shown by **7**, its second reduction was also able to be probed spectroelectrochemically (Figure 4d) and the spectrum of **7^2−^** observed was consistent with a dianionic NDI chromophore [21]. This behaviour confirms that triad **7** was stable under the conditions of the electrochemical experiment, showing superior stability to the single donor species triads, **3** or **4**, in both oxidation and reduction pathways.

**Figure 5:**
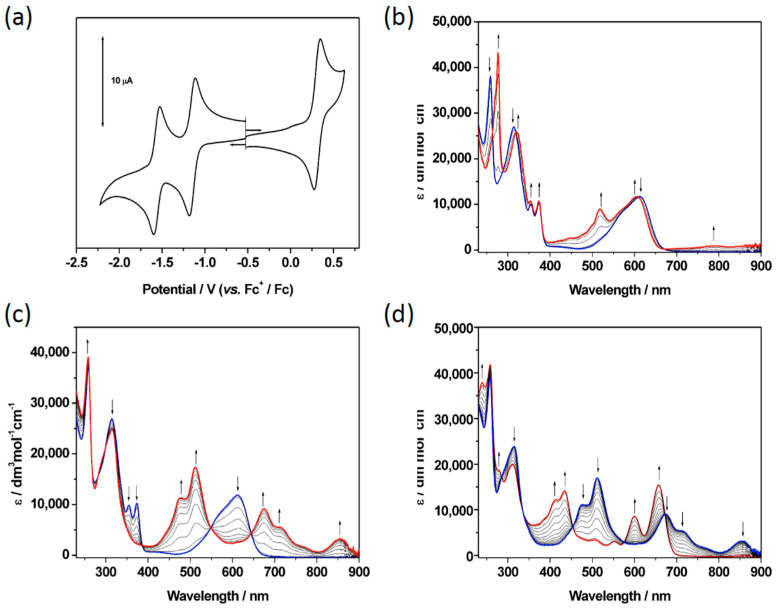
(**a**) Cyclic voltammogram of **8** and (**b**–**d**) UV/vis absorption spectra of **8** recorded in CH_2_Cl_2_ containing [NBu_4_][BF_4_] (0.4 M) using spectroelectrochemical methods at 243 K showing (**b**) the inter-conversion of **8** (blue) to **8^+^** (red) to the regenerated species (green); (**c**) the inter-conversion of **8** (blue) to **8^−^** (red); (**d**) the inter-conversion of **8^−^** (blue) to **8^2−^** (red). Arrows show the progress of (**b**) the oxidation and (**c**/**d**) the reduction.

For neutral molecules **8** and **9**, their absorption bands are red-shifted relative to their triad analogues, **3**, **4** and **7** (Table S1), due to the more electron donating nature of the morpholine substituent. Following oxidation of both **8** (Figure 5b) and **9** (Figure S31), the higher energy bands are depleted and replaced by red-shifted absorptions related to the oxidised species. Most notably a large additional peak is observed in the visible range at around 515 nm, however both compounds display a very low-level absorbance across the entire visible range. Viewing the initial reductions of both **8** (Figure 5c) and **9** (Figure S31), the band at ca. 600 nm is seen to deplete and new bands associated with the reduced species develop. The spectral profile of the anion also extends across the entire visible range, demonstrating the broad absorbing power of these hybrid amine-substituted NDI chromophores.

## 3. Conclusions

In this study, we report a strategy for the synthesis of donor–acceptor dyads and donor–acceptor–donor triads. Our approach employs core-functionalisation of NDI groups with amine groups that link the acceptor NDI with phenothiazine or phenoxazine donors. The dyads are based simply on core-linkage of NDI and phenothiazine/phenoxazine, **1** and **2**, but we are also able to functionalise the NDI with two such donor appendages leading to triad systems, **3** and **4**. Indeed, our strategy allowed sequential substitution with different appendages, e.g., phenothiazine followed by phenoxazine donors, making a hybrid donor–acceptor–donor triad, **7**. A similar strategy allows the modification of the NDI core by substitution with a different appendage, as in this case of an electron donating morpholino group, **8** and **9**. The electrochemical and spectroelectrochemical properties of these dyads and triads were explored, establishing that the donor phenothiazine/phenoxazine and acceptor NDI groups maintained characteristics of the parent functionality but could be incorporated into a single molecule. Our approach does influence the NDI properties due to amine substitution [2], but in each case, reversibility is observed for the reduction processes. Similarly, the phenothiazine or phenoxazine groups display reversible oxidation processes. In each case, the change in redox state is accompanied by the anticipated change in the UV/visible spectra of each component. For triad **7**, the oxidation processes of the phenothiazine and phenoxazine cannot be separated and a two-electron oxidation is observed between the oxidation potentials anticipated for each separate component. There is no evidence for direct coupling between the donors and acceptors in either dyad or triad with each component acting independently within the same molecule. Indeed, insight provided by elucidation of the solid-state structures of these compounds shows that the phenylene bridge between donor and acceptor moieties is out of plane of either unit, disrupting the conjugation of the molecule. As a result, these molecules provide promise for the development of charge separated systems [4] and lay the foundation for new avenues of research.

## Figures and Tables

**Table 1:** Reduction and oxidation potentials of **1**–**4**, **7**–**9** in CH_2_Cl_2_ containing [NBu_4_][BF_4_] (0.4 M) as the supporting electrolyte. Potentials reported relative to the internal standard E_1/2_ Fc^+^/Fc at a scan rate of 0.1 Vs^−1^ at room temperature. Values in brackets are ΔE (=E_p_^a^ − E_p_^c^). ΔE for the Fc^+^/Fc couple was 0.07 V under these conditions.

Compound	1st Ox E_1/2/_V	1st Red E_1/2/_V	2nd Red E_1/2/_V
**1**	+0.33 (0.07)	−1.06 (0.07)	−1.48 (0.07)
**2**	+0.36 (0.07)	−1.04 (0.07)	−1.48 (0.07)
**3**	+0.31 (0.08)	−1.14 (0.08)	−1.52 (0.08)
**4**	+0.33 (0.08)	−1.12 (0.07)	−1.51 (0.07)
**7**	+0.32 (0.08)	−1.12 (0.07)	−1.51 (0.07)
**8**	+0.31 (0.07)	−1.15 (0.07)	−1.56 (0.07)
**9**	+0.33 (0.07)	−1.14 (0.07)	−1.54 (0.07)
POZ-NH_2_	+0.25 (0.08)		
PTZ-NH_2_	+0.21 (0.07)		

## Supplementary Materials

The following supporting information can be downloaded at: https://www.mdpi.com/article/10.3390/molecules27248671/s1, Electronic supplementary information (ESI) available: Synthetic Methods, details of electrochemical and spectroelectrochemical measurements. Additional details of crystallographic studies. CCDC 2176091 (1), 2176092 (2), 2176093 (5), 2176094 (9) contain the supplementary crystallographic data for this paper [32,33,34]. These data can be obtained free of charge from The Cambridge Crystallographic Data Centre via www.ccdc.cam.ac.uk/data_request/cif (accessed on 30 November 2022).
