# Supplementary material for: Donor-Acceptor Dyads and Triads Employing Core-Substituted Naphthalene Diimides: A Synthetic and Spectro (Electrochemical) Study
Source: Molecules. 2022 Dec 8;27(24):8671. doi: 10.3390/molecules27248671 (PMC9783862; doi:10.3390/molecules27248671)
Supplement: Supplementary file 1 [file molecules-27-08671-s001.zip › molecules-2049795-supplementary.pdf]

molecules

Supplementary Material

Donor-Acceptor Dyads and Triads Employing Core-Substituted Naphthalene Diimides: A Synthetic and Spectro (Electrochemical) Study

Samuel Quinn [1], E. Stephen Davies [1], Nicholas Pearce [2], Callum Rosenberg [2], Constance R. Pfeiffer [1], Georgia R. F. Orton [2] and Neil R. Champness [2,*]

Supporting Information Table of Contents

Experimental Details including Synthesis	Page 1
NMR Spectra	Page 8
Details of X-ray Crystallographic Experiments	Page 19
Details of Electrochemical and Optical Investigations	Page 21
Additional UV-vis Spectra and Spectroelectrochemistry and Data	Page 25
References	Page 28

Experimental

General procedures

All starting materials were purchased form Sigma Aldrich or Fisher Scientific and used without further purification. Column chromatography was conducted using silica gel (Merck silica gel 60, 0.2-0.5 mm, 50-130 mesh). The ^{1}H and ^{13}C NMR spectra were obtained on a Bruker 300, 400 or 500 MHz spectrometers. MALDI MS spectra were collected with a Bruker Ultraflex III mass spectrometer using trans-2-[3-(4-tert-Butylphenyl)-2-methyl-2-propenylidene]malononitrile as the matrix. FD MS spectra were measured with a JOEL AccuTOF GCX spectrometer.

Brominated NDIs were prepared by literature methods.[S1,2] 10-(4-aminophenyl)-10H-phenothiazine (PTZ-NH_2),[S3] was initially prepared according to literature methods but an improved synthetic path was also developed as described below. 10-(4-aminophenyl)-10H-phenothiazine slowly decomposed in light at room temperature and so was stored in the dark at -18 °C.

Alternative synthesis of 10-(4-aminophenyl)-10H-phenothiazine (PTZ-NH_2)

10-(4-nitrophenyl)-10H-phenothiazine (700 mg, 2.17 mmol) and Pd/C 10% (245 mg, 0.22 mmol) were added to THF (25 mL) and stirred under an atmosphere of H_2 at RT, in the dark for 16 h. The resulting solution was filtered through kieselguhr, the solvent removed and the residue purified *via* column chromatography (silica, $CHCl_3$:Hexane [3:1]) to give 10-(4-aminophenyl)-10H-phenothiazine (402 mg, 1.39 mmol, 64%) as a white powder. ^{1}H NMR (400 MHz, Chloroform-*d*) δ 7.18 (d, *J* = 8.6 Hz, 2H), 7.01 (dd, *J* = 7.4, 1.7 Hz, 2H), 6.93 – 6.77 (m, 6H), 6.29 (dd, *J* = 8.1, 1.4 Hz, 2H), 3.87 (s, 2H) ppm. ^{13}C NMR (101 MHz, Chloroform-*d*) δ 146.31, 144.84, 132.04, 131.19, 126.78, 126.53, 122.08, 119.51, 116.73, 115.64 ppm. ESI MS: Found: 290.103 [M^+] Calc: 290.088.

Synthesis of 4-nitrophenylboronic acid

To dry DMF (90 mL), degassed with N_2 was added 4-iodonitrobenzene (3.81 g, 15.3 mmol), bis(pinacolato)diboron (5.45 g, 21.5 mmol), [1,1'-Bis(diphenylphosphino)ferrocene]palladium(II) dichloride (330 mg, 0.451 mmol) and potassium acetate (4.51 g, 46 mmol). The mixture was heated to 70 °C for 2 h, cooled to RT and water (200 mL) was added. The aqueous layer was extracted with diethyl ether (4 x 100 mL) and the ethereal phase was further washed with water (100 mL), brine (100 mL), dried over $MgSO_4$ and filtered. The solvent was removed and the residue was dissolved in THF (12 mL) and water (12 mL). Sodium periodate (9.85 g, 46.2 mmol) was added and the solution stirred at room temperature for 1.5 h. HCl (1 M, 35 mL) was added and the solution stirred for 3 h then extracted into ethyl acetate (4 x 50 mL) and the organic phase was washed with brine, dried over $MgSO_4$ and filtered. The solvent was removed and the residue was dissolved in sodium hydroxide solution (0.5 M, 50 mL), filtered and stirred over activated charcoal for 30 min. The resulting solution was filtered, cooled in an ice bath and acidified dropwise with conc. HCl. The precipitate was collected to give of 4-nitrophenylboronic acid (1.87 g, 11.2 mmol, 73%) as a white powder. ^{1}H NMR (400 MHz, Chloroform-*d*) δ 8.23 (d, *J* = 8.7 Hz, 2H), 7.94 (d, *J* = 8.5 Hz, 2H), 5.02 (brs, 1H) ppm. ^{13}C NMR (101 MHz, *DMSO-d6*) δ data: 149.2, 135.4, 122.5 ppm. ESI MS: Found: 167.057 [M^+] Calc: 167.039.

Synthesis of 10-(4-nitrophenyl)-10H-phenoxazine (POZ-NO_2)

Phenoxazine (100 mg, 550 µmol), 4-nitrophenylboronic acid (230 mg, 1.38 mmol) and copper acetate (150 mg, 830 µmol) were combined in dry degassed dichloromethane (5 mL). To this dry pyridine (90 µL, 1.1 mmol) was added. The solution was then exposed to air and left to stir at RT for 18 h. The

solution was filtered through kieselguhr and washed with HCl (2 M, 100 ml) and brine (100 ml), then dried over $MgSO_4$, filtered and the solvent removed. The residue was purified *via* column chromatography (silica, DCM:Hexane [7:3]) to give 10-(4-nitrophenyl)-10H-phenoxazine (65 mg, 0.197 mmol, 39%) as an orange powder. ^{1}H NMR (400 MHz, Chloroform-*d*) δ 8.46 (d, *J* = 8.7 Hz, 2H), 7.58 (d, *J* = 8.7 Hz, 2H), 6.80 – 6.73 (m, 4H), 6.66 (td, *J* = 7.5, 2.1 Hz, 2H), 6.02 (d, *J* = 7.9 Hz, 2H) ppm. ^{13}C NMR (101 MHz, Chloroform-*d*) δ 145.74, 144.37, 133.09, 131.25, 126.38, 123.37, 122.57, 122.56, 116.08, 113.77 ppm. ESI MS: Found: 304.068 [M^+] Calc: 304.085.

Synthesis of 10-(4-aminophenyl)-10H-phenoxazine (POZ-NH_2)

10-(4-Nitrophenyl)-10H-phenoxazine (100 mg, 0.33 mmol) and Pd/C 10% (7 mg, 66 µmol) were added to THF (5 mL) and stirred under an atmosphere of hydrogen at RT, in the dark for 16 h. The resulting solution was filtered through kieselguhr, the solvent removed and the residue purified *via* column chromatography (silica, $CHCl_3$:Hexane [3:1]) to give 10-(4-aminophenyl)-10H-phenoxazine (63 mg, 0.230 mmol, 70%) as a grey powder. ^{1}H NMR (300 MHz, Chloroform-*d*) δ 7.15 – 7.03 (m, 2H), 6.91 – 6.80 (m, 2H), 6.69 – 6.55 (m, 6H), 6.04 – 5.92 (m, 2H), 3.84 (s, 2H) ppm. ^{13}C NMR (75 MHz, Chloroform-*d*) δ 146.41, 143.98, 134.95, 131.49, 129.10, 123.16, 120.87, 116.97, 115.17, 113.21 ppm. ESI MS: Found: 274.136 [M^+] Calc: 274.111.

Synthesis of 1

2-Bromo-*N,N'*-bis(2,6-diisopropylphenyl)naphthalene-1,4,5,8-tetracarboxylic diimide (25 mg, 38 µmol) and 10-(4-nitrophenyl)-10H-phenothiazine (11 mg, 38 µmol) were dissolved in DMF (1 mL), and the solution was heated to 135 °C for 4 h. This resulting mixture was cooled, the solvent removed and the residue extracted with $CHCl_3$ (20 mL), washed with HCl (2 M, 2 x 20 mL), dried over $MgSO_4$, filtered, the solvent was removed and the residue purified *via* column chromatography (silica, $CHCl_3$:EtOAc [9:1]) to give **1** (29 mg, 33 µmol, 86%) as a pink powder. ^{1}H NMR (400 MHz, Chloroform-*d*) δ 11.95 (s, 1H), 8.86 (d, *J* = 7.8 Hz, 1H), 8.84 (s, 1H), 8.57 (d, *J* = 7.8 Hz, 1H), 7.58 – 7.49 (m, 4H), 7.48 – 7.43 (m, 2H), 7.40 (d, *J* = 7.7 Hz, 2H), 7.35 (d, *J* = 7.8 Hz, 2H), 7.09 (dd, *J* = 7.5, 1.7 Hz, 2H), 6.99 – 6.92 (m, 2H), 6.88 (td, *J* = 7.5, 1.3 Hz, 2H), 6.44 (dd, *J* = 8.1, 1.3 Hz, 2H), 2.73 (dp, *J* = 16.8, 6.8 Hz, 4H), 1.22 (dd, *J* = 6.8, 4.1 Hz, 12H), 1.18 (d, *J* = 6.8 Hz, 12H) ppm. ^{13}C NMR (101 MHz, Chloroform-d) δ 166.74, 163.34, 162.87, 162.84, 150.19, 145.52, 145.45, 143.76, 139.86, 136.51, 132.30, 130.70, 130.50, 130.30, 130.02, 129.92, 129.88, 128.30, 127.11, 127.05, 126.62, 126.18, 125.55, 124.50, 124.30, 124.19, 123.15, 122.33, 121.73, 121.51, 117.49, 101.76, 29.27, 29.25, 24.04, 24.01, 23.99, 23.95 ppm. FD MS: 874.35601 [M^+] Calc: 874.35528.

Synthesis of 2

2-Bromo-*N,N′*-bis(2,6-diisopropylphenyl)naphthalene-1,4,5,8-tetracarboxylic diimide (25 mg, 38 µmol) and 10-(4-nitrophenyl)-10H-phenoxazine (11 mg, 38 µmol) were dissolved in DMF (1 mL and the solution was heated to 135 °C for 4 h. This resulting mixture was cooled, the solvent removed and the residue extracted with $CHCl_3$ (20 mL), washed with HCl (2 M, 2 x 20 mL), dried over $MgSO_4$, filtered, the solvent was removed and the residue purified *via* column chromatography (silica, $CHCl_3$:EtOAc [9:1]) to give **2** (20 mg, 24 µmol, 62%) as a pink powder. ^{1}H NMR (400 MHz, Chloroform-*d*) δ 11.95 (s, 1H), 8.87 (d, *J* = 7.8 Hz, 1H), 8.83 (s, 1H), 8.59 (d, *J* = 7.8 Hz, 1H), 7.63 – 7.58 (m, 2H), 7.56 – 7.49 (m, 2H), 7.48 – 7.44 (m, 2H), 7.41 (d, *J* = 7.8 Hz, 2H), 7.35 (d, *J* = 7.8 Hz, 2H), 6.73 – 6.60 (m, 6H), 6.00 (dd, *J* = 7.5, 1.7 Hz, 2H), 2.73 (dp, *J* = 16.9, 6.8 Hz, 4H), 1.22 (dd, *J* = 6.9, 4.5 Hz, 12H), 1.18 (d, *J* = 6.8 Hz, 12H) ppm. ^{13}C NMR (101 MHz, Chloroform-*d*) δ 166.76, 163.24, 162.84, 162.83, 150.08, 145.52, 145.43, 143.93, 137.65, 137.12, 134.07, 132.69, 132.35, 130.46, 130.26, 129.99, 129.94, 129.90, 128.34, 126.64, 126.30, 126.01, 124.54, 124.30, 124.19, 123.37, 121.60, 121.54, 115.55, 113.27, 101.95, 29.28, 29.26, 24.05, 24.02, 24.00, 23.95 ppm. FD MS: Found: 858.37867 Calc[M^+]: 858.37812.

Synthesis of 5

2,6-Dibromo-*N,N′*-bis(2,6-diisopropylphenyl)naphthalene-1,4,5,8-tetracarboxylic diimide (200 mg, 269 µmol) and 10-(4-nitrophenyl)-10H-phenothiazine (117 mg, 404 µmol) were dissolved in DMF (5 mL) and heated to 135 °C for 4 h. The solvent was removed and the residue purified *via* column chromatography (alumina, DCM:Hexane [6:4]) to give **5** (119 mg, 125 µmol, 47%) as a pink powder. A small quantity of a blue powder, **3** (23 mg, 19 µmol, 7%) can also be isolated from this reaction, however, it is best to prepare **3** by further reaction of **5** with PTZ-NH_2, described below. ^{1}H NMR (400 MHz, Chloroform-*d*) δ 11.90 (s, 1H), 9.07 (s, 1H), 8.89 (s, 1H), 7.59 – 7.43 (m, 6H), 7.40 (d, *J* = 7.8 Hz, 2H), 7.34 (d, *J* = 7.8 Hz, 2H), 7.10 (dd, *J* = 7.5, 1.7 Hz, 2H), 6.96 (td, *J* = 7.7, 1.7 Hz, 2H), 6.89 (td, *J* = 7.5, 1.3 Hz, 2H), 6.45 (dd, *J* = 8.0, 1.3 Hz, 2H), 2.79 – 2.63 (m, 4H), 1.22 (dd, *J* = 6.8, 3.7 Hz, 12H), 1.18 (dd, *J* = 6.8, 2.3 Hz, 12H) ppm. ^{13}C NMR (126 MHz, Chloroform-*d*) δ 166.46, 161.82, 161.80, 161.36, 149.70, 145.47, 145.32, 143.69, 140.08, 139.21, 136.22, 130.48, 130.11, 130.03, 129.96, 129.91, 129.57, 127.90, 127.15, 127.05, 125.51, 124.35, 124.22, 124.19, 124.15, 123.55, 123.22, 122.56, 122.33, 122.31, 117.65, 101.76, 29.32, 29.30, 24.06, 24.03, 23.97, 23.93 ppm. MALDI MS: Found: 952.29 Calc[M^+]: 952.26.

Synthesis of 3

5, (25 mg, 26 µmmol) and 10-(4-nitrophenyl)-10H-phenothiazine (24 mg, 83 µmol) were dissolved in DMF (1 mL) and heated to 135 °C for 16 h. The mixture was extracted with $CHCl_3$ (50 mL) and washed with HCl (2 M, 2 x 100 mL), brine (100 mL), dried over $MgSO_4$, filtered and the solvent removed. The residue was purified *via* column chromatography (alumina, $CHCl_3$:Hexane [1:1]) to give **3** as a blue powder (15 mg, 13 µmol, 49%). ^{1}H NMR (500 MHz, Benzene-*d6*:Chloroform-*d* [0.6:0.4]) δ 11.34 (s, 2H), 8.83 (s, 2H), 7.36 – 7.32 (m, 2H), 7.22 (d, *J* = 7.9 Hz, 4H), 7.18 (d, *J* = 8.6 Hz, 4H), 7.01 – 6.98 (m, 4H), 6.90 (dd, *J* = 7.5, 1.6 Hz, 4H), 6.72 – 6.68 (m, 4H), 6.62 (td, *J* = 7.5, 1.3 Hz, 4H), 6.18 (dd, *J* = 8.2, 1.3 Hz, 4H), 2.80 (p, *J* = 6.8 Hz, 4H), 1.17 (dd, *J* = 8.2, 6.8 Hz, 24H) ppm. ^{13}C NMR (126 MHz, Benzene-*d6*:Chloroform-*d* [0.6:0.4]) δ 167.00, 162.85, 147.09, 145.79, 144.42, 138.57, 138.03, 132.01, 130.71, 130.13, 127.17, 126.93, 126.68, 124.84, 124.49, 123.74, 123.03, 121.51, 121.15, 116.90, 104.05, 29.65, 24.32, 24.28 ppm. FD MS: Found: 1162.42718 Calc[M$^+$]: 1162.42740.

Synthesis of 6

2,6-Dibromo-*N,N'*-bis(2,6-diisopropylphenyl)naphthalene-1,4,5,8-tetracarboxylic diimide (120 mg, 170 µmol) and 10-(4-nitrophenyl)-10H-phenoxzine (60 mg, 0.220 µmol) were dissolved in DMF (2 mL) and heated to 135 °C for 4 h. The solvent was removed and the residue purified *via* column chromatography (silica, $CHCl_3$:EtOAc [9:1]) to give **6** (141 mg, 150 µmol, 89%) as a pink powder. ^{1}H NMR (500 MHz, Chloroform-*d*) δ 11.91 (s, 1H), 9.07 (s, 1H), 8.88 (s, 1H), 7.62 – 7.57 (m, 2H), 7.56 (t, *J* = 7.8 Hz, 1H), 7.50 (t, *J* = 7.8 Hz, 1H), 7.48 – 7.44 (m, 2H), 7.41 (d, *J* = 7.8 Hz, 2H), 7.35 (d, *J* = 7.8 Hz, 2H), 6.70 (td, *J* = 7.3, 1.7 Hz, 2H), 6.68 – 6.61 (m, 4H), 5.99 (dd, *J* = 7.7, 1.6 Hz, 2H), 2.71 (dp, *J* = 15.7, 6.9 Hz, 4H), 1.22 (dd, *J* = 6.9, 5.4 Hz, 12H), 1.18 (dd, *J* = 6.8, 2.5 Hz, 12H) ppm. ^{13}C NMR (126 MHz, Chloroform-*d*) δ 166.49, 161.81, 161.78, 161.34, 149.58, 145.47, 145.31, 143.93, 139.27, 137.47, 137.27, 134.03, 132.74, 130.08, 130.05, 129.94, 129.92, 129.53, 127.94, 125.99, 124.36, 124.24, 124.23, 124.18, 123.59, 123.37, 122.50, 122.19, 121.62, 115.57, 113.26, 101.98, 29.32, 29.31, 24.06, 24.04, 23.98, 23.93 ppm. FD MS: Found: 936.28882 Calc[M$^+$]: 936.28863.

Synthesis of 4

6, (16 mg, 17 µmmol) and 10-(4-nitrophenyl)-10H-phenoxazine (51 mg, 186 µmol) were dissolved in DMF (1 mL) and heated to 135 °C for 16 h. The mixture was extracted with $CHCl_3$ (50 mL) and washed with HCl (2 M, 2 x 100 mL), brine (100 mL), dried over $MgSO_4$, filtered and the solvent removed. The residue was purified *via* column chromatography (silica, DCM:Hexane [7:3]) to give **4** as a blue powder (17 mg, 15 µmol, 88%). ^{1}H NMR (500 MHz, Methylene Chloride-*d2*:Benzene-*d6*:Chloroform-

d [0.7:0.2:0.1]) δ 11.60 (s, 2H), 9.07 (s, 2H), 7.75 – 7.68 (m, 6H), 7.57 (d, *J* = 7.7 Hz, 4H), 6.90 (dt, *J* = 7.9, 4.0 Hz, 4H), 6.82 (qt, *J* = 7.4, 3.8 Hz, 12H), 6.23 (dt, *J* = 8.1, 4.0 Hz, 4H), 3.02 (p, *J* = 7.0 Hz, 4H), 1.42 (dd, *J* = 10.6, 6.8 Hz, 24H) ppm. ^{13}C NMR (126 MHz, Methylene Chloride-*d2*:Benzene-*d6*:Chloroform-*d* [0.7:0.2:0.1]) δ 166.42, 162.34, 146.57, 145.61, 143.75, 138.43, 135.70, 134.06, 132.09, 130.48, 129.43, 126.14, 124.99, 123.90, 123.19, 123.08, 121.14, 120.61, 115.08, 113.11, 103.66, 29.47, 23.57, 23.52 ppm. FD MS: Found: 1130.47356 Calc[M$^+$]: 1130.47308.

Synthesis of 7

5 (50 mg, 52 µmol) and 10-(4-nitrophenyl)-10H-phenoxazine (15 mg, 52 µmol) were dissolved in DMF (1 mL) and heated to 135 °C for 2 h. the solvent was removed and the residue purified *via* column chromatography (silica, $CHCl_3$:EtOAc [19:1]) to give **7** (28 mg, 24 µmol, 46%) as a blue powder. ^{1}H NMR (500 MHz, Methylene Chloride-*d2*) δ 11.36 (s, 1H), 11.35 (s, 1H), 8.86 (s, 1H), 8.85 (s, 1H), 7.65 – 7.61 (m, 4H), 7.52 (d, *J* = 7.7 Hz, 2H), 7.48 – 7.45 (m, 2H), 7.45 – 7.43 (m, 2H), 7.38 (d, *J* = 7.8 Hz, 4H), 7.04 (dd, *J* = 7.6, 1.6 Hz, 2H), 6.92 (ddd, *J* = 8.2, 7.4, 1.6 Hz, 2H), 6.86 – 6.83 (m, 2H), 6.68 – 6.66 (m, 2H), 6.63 (ddd, *J* = 7.0, 4.8, 2.0 Hz, 4H), 6.41 (dd, *J* = 8.1, 1.3 Hz, 2H), 6.06 – 6.04 (m, 2H), 2.79 – 2.73 (m, 4H), 1.19 (d, *J* = 6.8 Hz, 12H), 1.17 (d, *J* = 6.9 Hz, 12H) ppm. ^{13}C NMR (126 MHz, Methylene Chloride-*d2*) δ 166.20, 166.19, 162.19, 162.18, 146.50, 146.36, 145.45, 143.66, 143.48, 138.41, 137.89, 137.74, 135.44, 133.87, 131.92, 131.08, 130.28, 129.19, 127.49, 127.30, 127.10, 126.53, 126.30, 125.95, 125.94, 124.88, 124.57, 123.70, 123.00, 122.95, 122.88, 122.30, 120.90, 120.69, 120.49, 120.42, 116.32, 114.82, 112.88, 103.52, 103.40, 29.22, 28.72, 23.33, 23.28, 22.22, 22.18 ppm. FD MS: Found: 1146.45047 Calc[M$^+$]: 1146.45024.

Synthesis of 8

5 (50 mg, 53 µmol) and morpholine (10 µL, 110 µmol) were dissolved in DMF (0.5 mL) and heated to 135 °C for 2 h. The solvent was removed and the residue purified *via* column chromatography (silica, $CHCl_3$:EtOAc [19:1]) to give **8** (27 mg, 28 µmol, 52%) as a blue powder. ^{1}H NMR (400 MHz, Chloroform-*d*) δ 11.54 (s, 1H), 8.88 (s, 1H), 8.56 (s, 1H), 7.58 – 7.46 (m, 4H), 7.42 (dd, *J* = 12.6, 8.2 Hz, 4H), 7.34 (d, *J* = 7.8 Hz, 2H), 7.06 (dd, *J* = 7.4, 1.7 Hz, 2H), 6.91 (dd, *J* = 7.9, 1.7 Hz, 2H), 6.85 (td, *J* = 7.4, 1.4 Hz, 2H), 6.37 (dd, *J* = 8.1, 1.3 Hz, 2H), 3.95 (t, *J* = 4.5 Hz, 4H), 3.43 (t, *J* = 4.6 Hz, 4H), 2.73 (dp, *J* = 20.7, 6.9 Hz, 4H), 1.22 (dd, *J* = 6.9, 2.7 Hz, 12H), 1.17 (t, *J* = 6.2 Hz, 12H) ppm. ^{13}C NMR (101 MHz, Chloroform-*d*) δ 166.70, 162.75, 162.73, 161.78, 151.95, 147.37, 145.51, 145.34, 143.99, 138.60, 137.69, 131.42, 130.84, 130.35, 129.87, 129.68, 127.58, 127.02, 126.94, 125.53, 125.24, 124.88, 124.76, 124.34,

124.27, 124.15, 122.86, 121.42, 121.01, 116.85, 66.78, 52.61, 29.25, 24.10, 24.05, 24.01, 23.91 ppm. FD MS: Found: 959.40831 Calc[M^+]: 959.40804.

Synthesis of 9

6 (50 mg, 53 µmol) and morpholine (10 µL, 110 µmol) were dissolved in DMF (0.5 mL) and heated to 135 °C for 2 h. The solvent was removed and the residue purified *via* column chromatography (silica, $CHCl_3$:EtOAc [19:1]) to give **9** (50 mg, 53 µmol, 99%) as a blue powder. ^{1}H NMR (400 MHz, Chloroform-*d*) δ 11.52 (s, 1H), 8.86 (s, 1H), 8.55 (s, 1H), 7.52 (dt, *J* = 20.4, 8.1 Hz, 4H), 7.39 (d, *J* = 8.1 Hz, 4H), 7.34 (d, *J* = 7.8 Hz, 2H), 6.70 – 6.58 (m, 6H), 5.99 (dd, *J* = 7.6, 1.7 Hz, 2H), 3.97 – 3.91 (m, 4H), 3.45 – 3.39 (m, 4H), 2.72 (dp, *J* = 20.6, 6.8 Hz, 4H), 1.22 (dd, *J* = 6.8, 2.7 Hz, 12H), 1.19 – 1.14 (m, 12H) ppm. ^{13}C NMR (101 MHz, Chloroform-*d*) δ 166.70, 162.74, 162.72, 161.77, 151.98, 147.27, 145.50, 145.32, 143.91, 138.41, 136.14, 134.18, 132.45, 130.82, 130.27, 129.88, 129.69, 127.58, 125.57, 125.22, 125.17, 124.83, 124.38, 124.27, 124.15, 123.36, 121.47, 120.95, 115.47, 113.28, 111.64, 102.97, 66.78, 52.60, 29.25, 24.10, 24.05, 24.01, 23.91 ppm. FD MS: Found: 943.43062 Calc[M^+]: 943.43088.

NMR Spectra

10-(4-nitrophenyl)-10H-phenoxazine

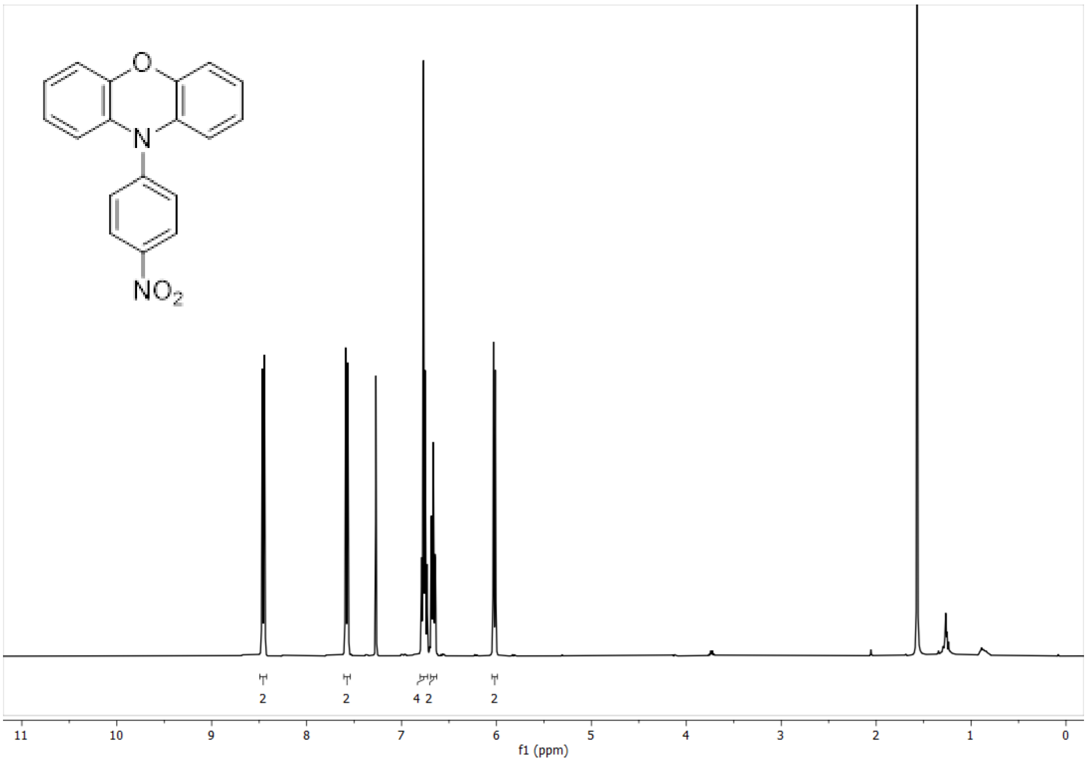

Figure S1. ^{1}H NMR Spectrum of 10-(4-nitrophenyl)-10H-phenoxazine.

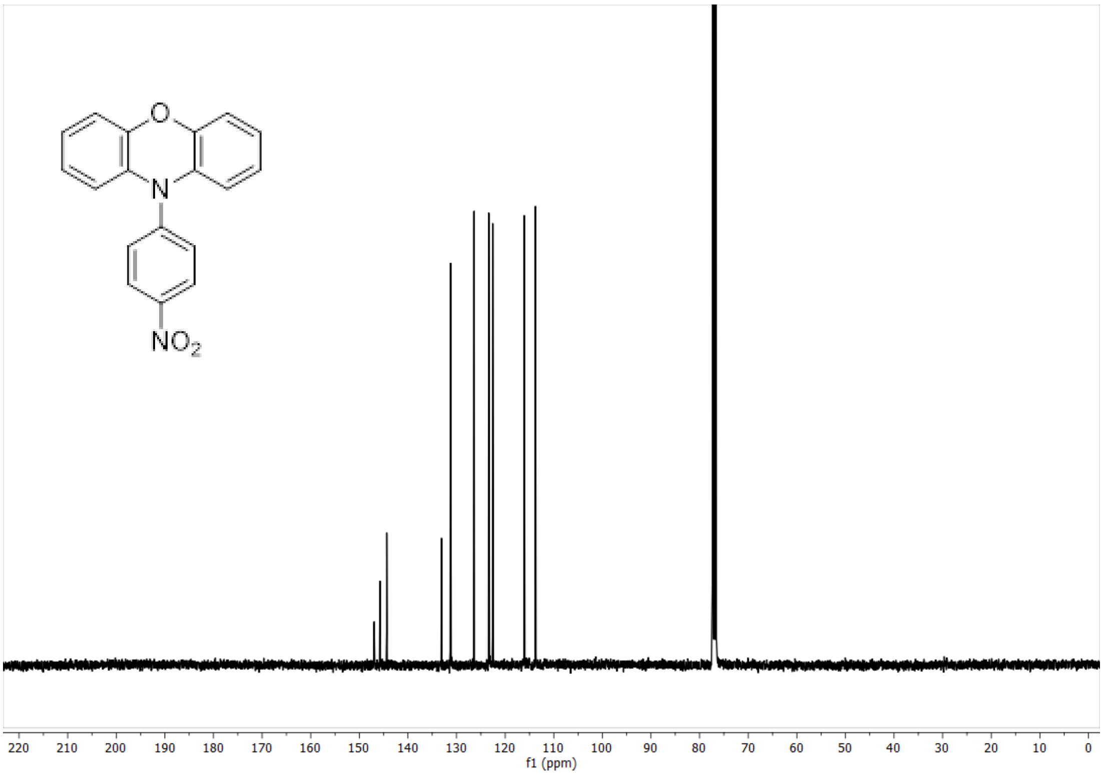

Figure S2. ^{13}C NMR Spectrum of 10-(4-nitrophenyl)-10H-phenoxazine.

10-(4-aminophenyl)-10H-phenoxazine

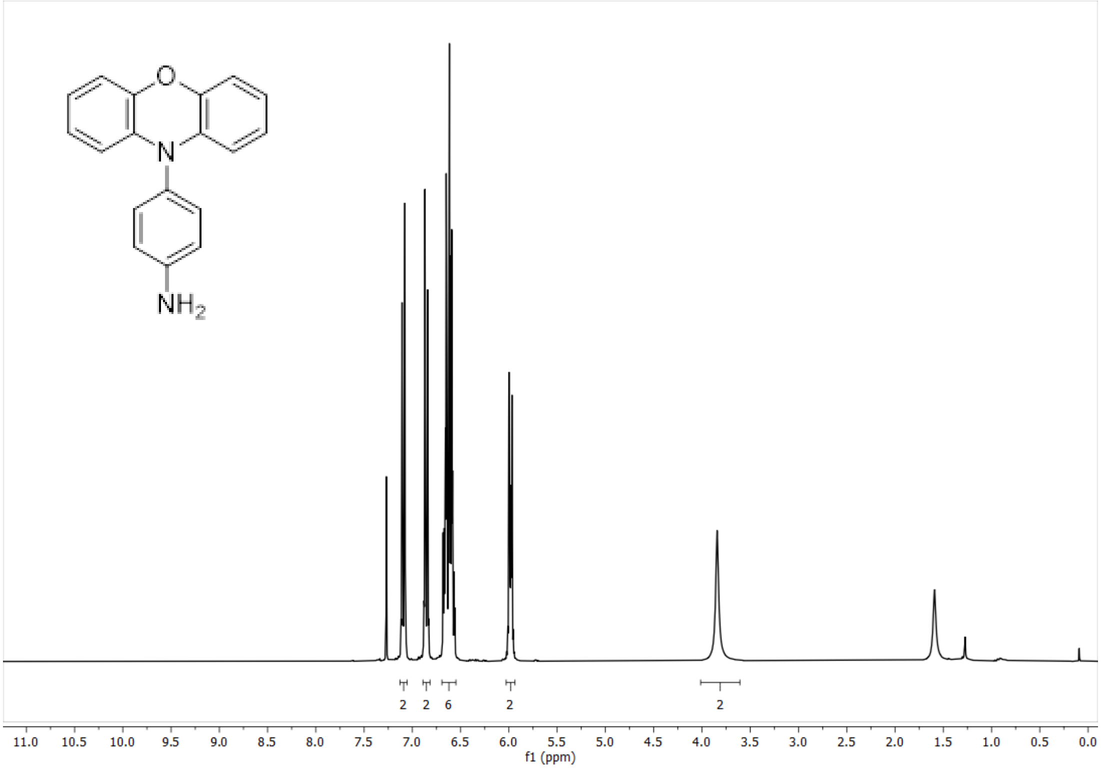

Figure S3. ^{1}H NMR Spectrum of 10-(4-aminophenyl)-10H-phenoxazine.

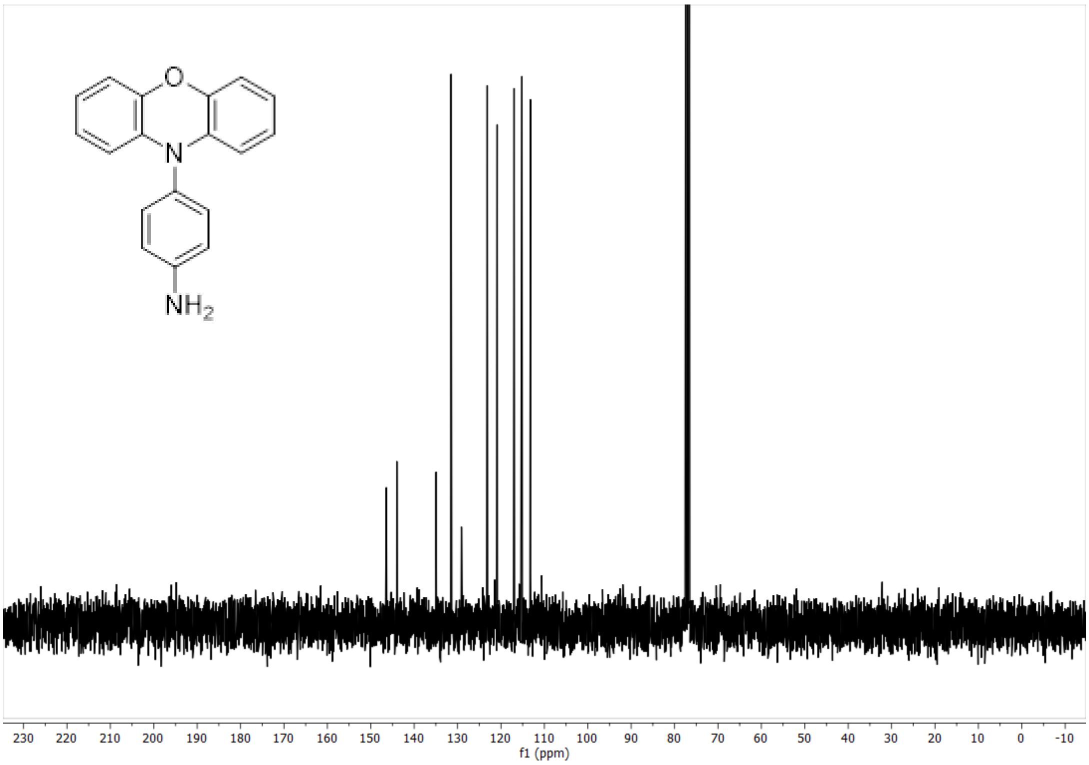

Figure S4. ^{13}C NMR Spectrum of 10-(4-aminophenyl)-10H-phenoxazine.

Compound **1**

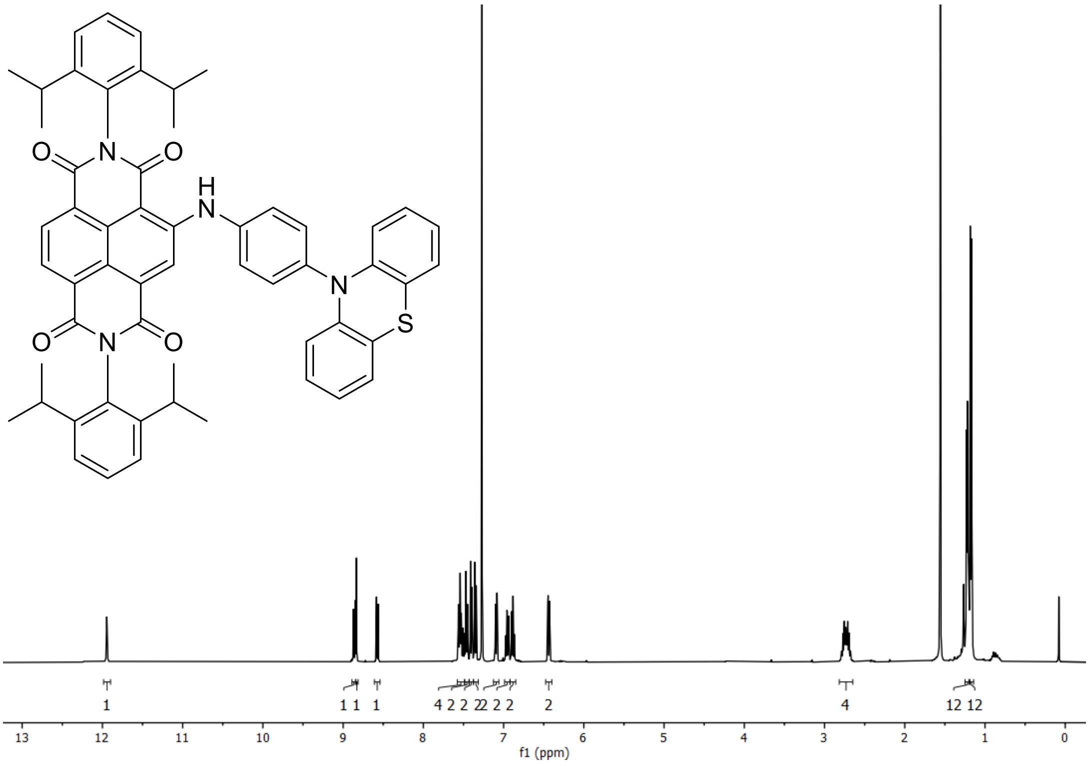

Figure S5. ^{1}H NMR Spectrum of compound **1**.

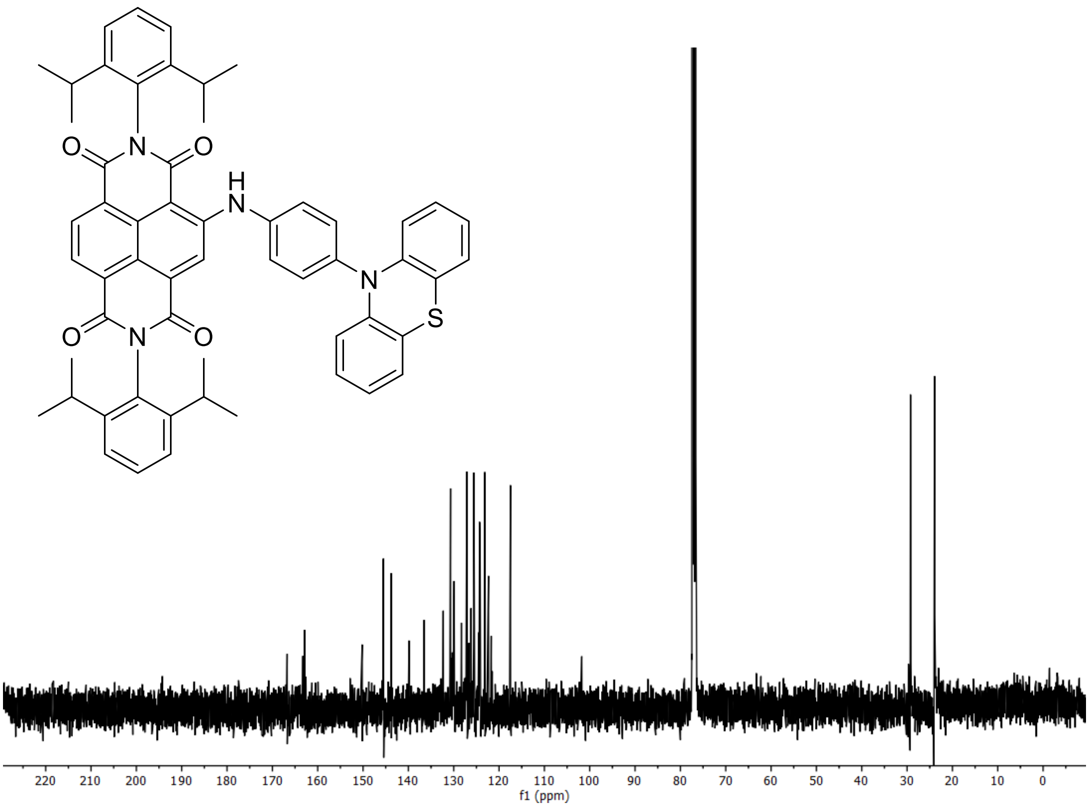

Figure S6. ^{13}C NMR Spectrum of compound **1**.

Compound **2**

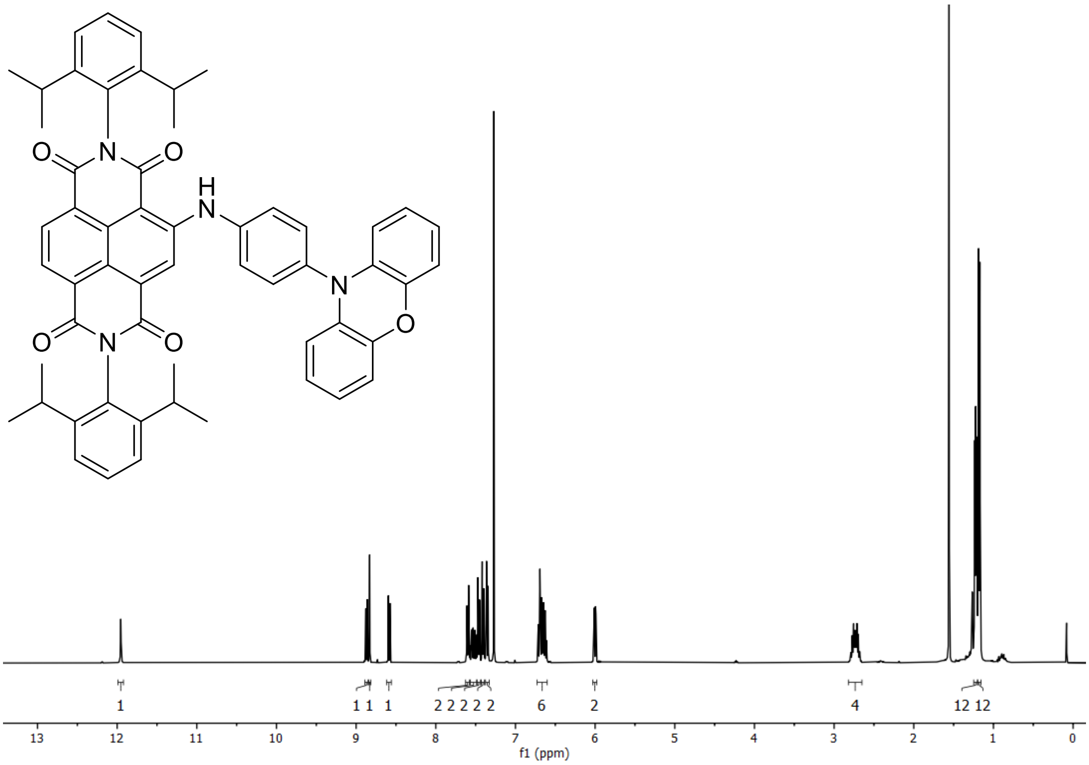

Figure S7. ^{1}H NMR Spectrum of compound **2**.

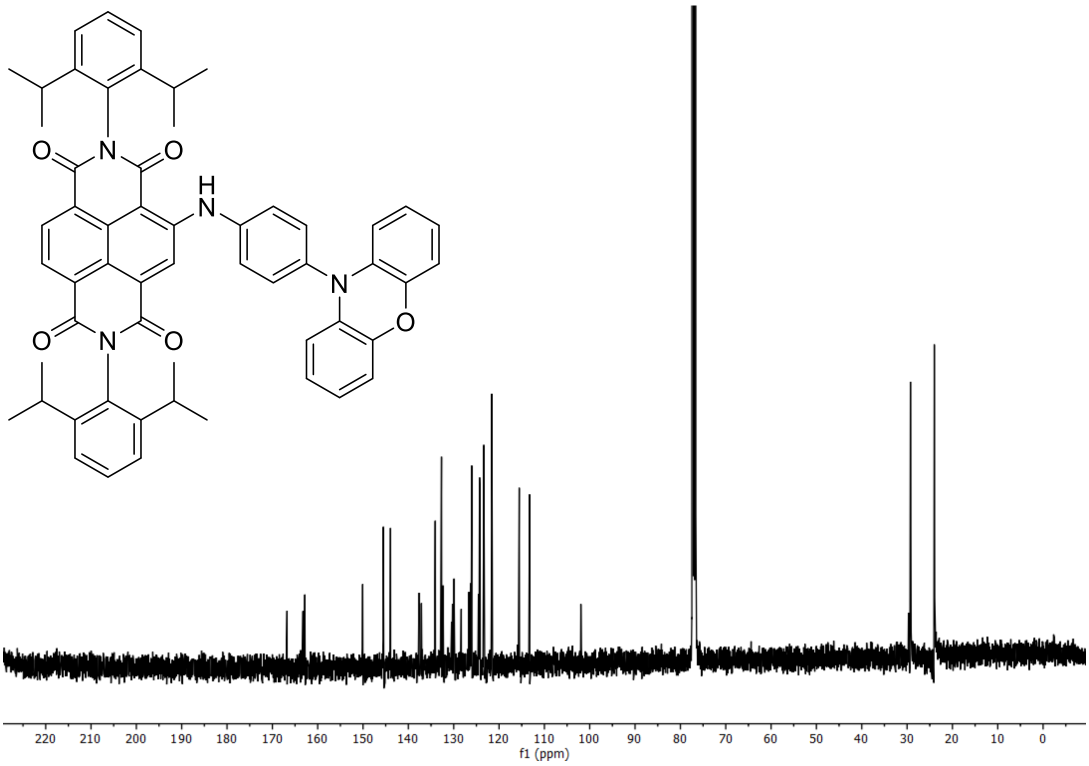

Figure S8. ^{13}C NMR Spectrum of compound **2**.

Compound **3**

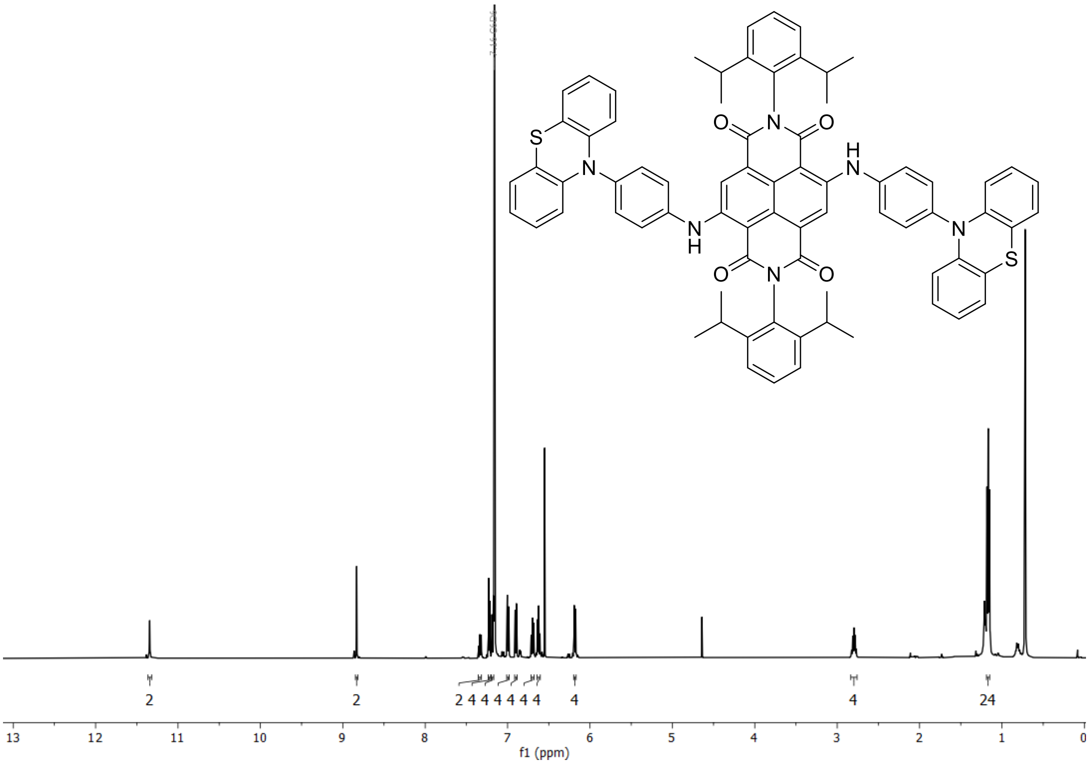

Figure S9. ^{1}H NMR Spectrum of compound **3**.

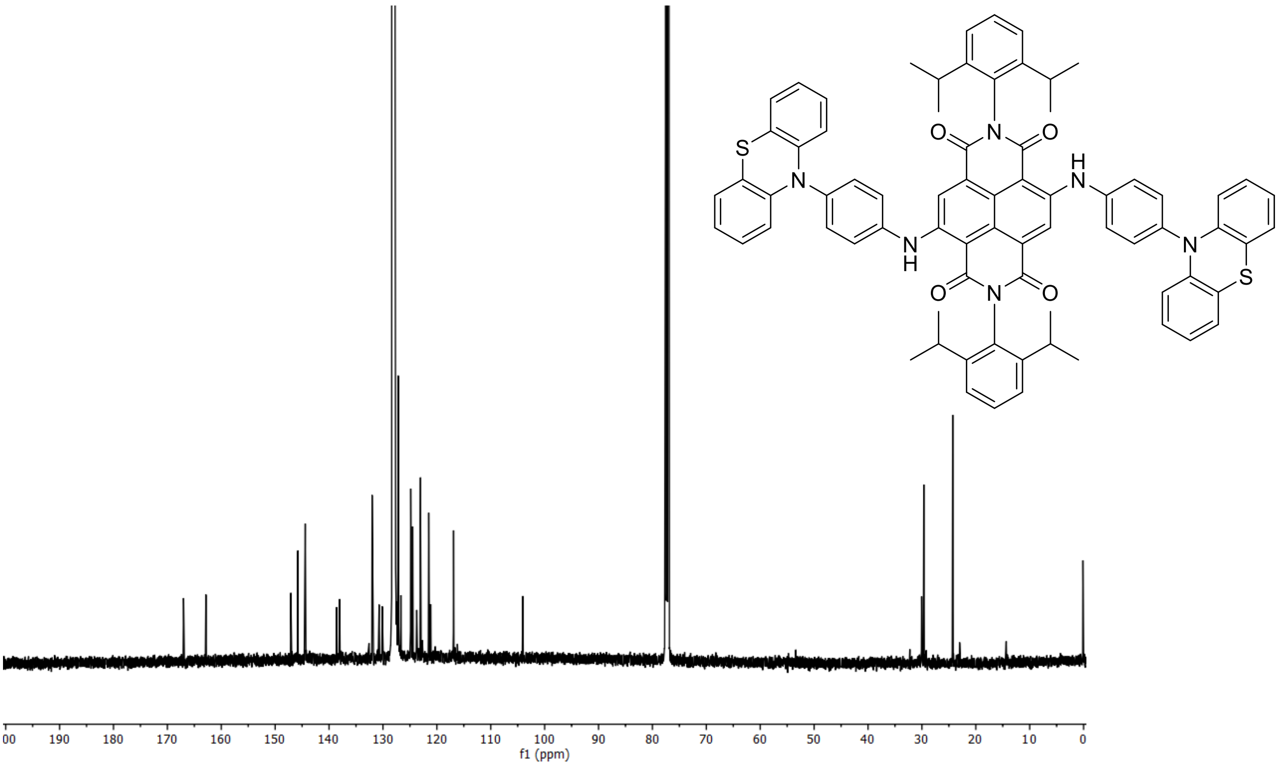

Figure S10. ^{13}C NMR Spectrum of compound **3**.

Compound **4**

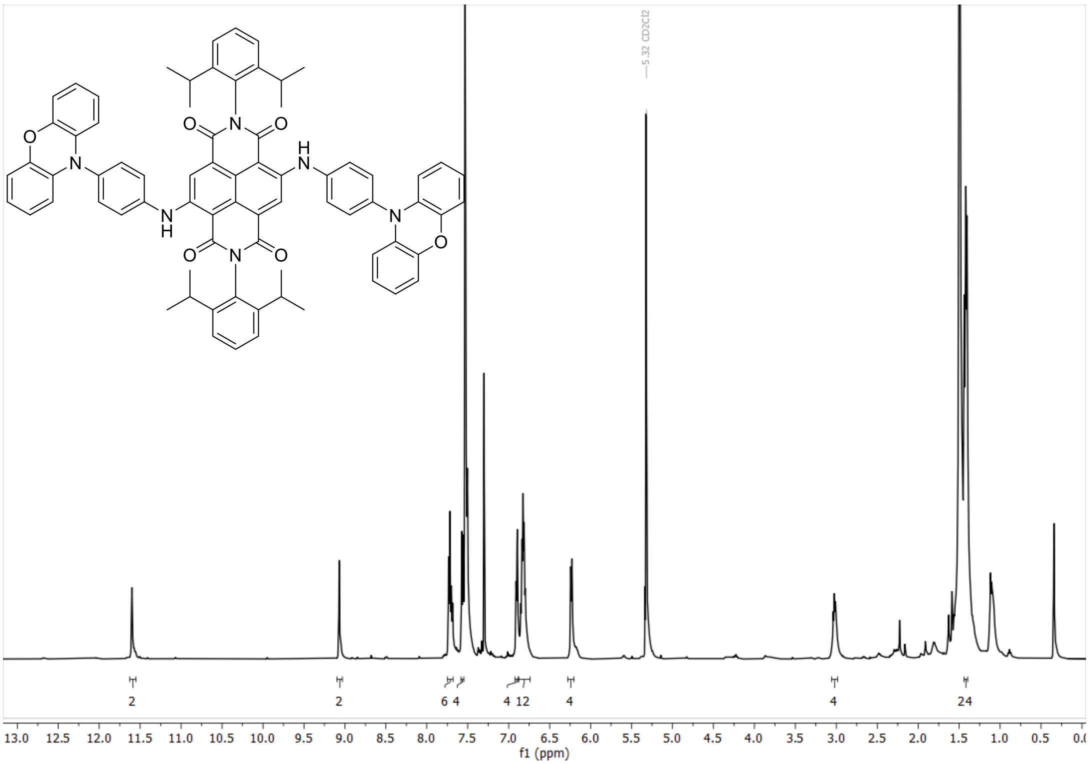

Figure S11. ^{1}H NMR Spectrum of compound **4**.

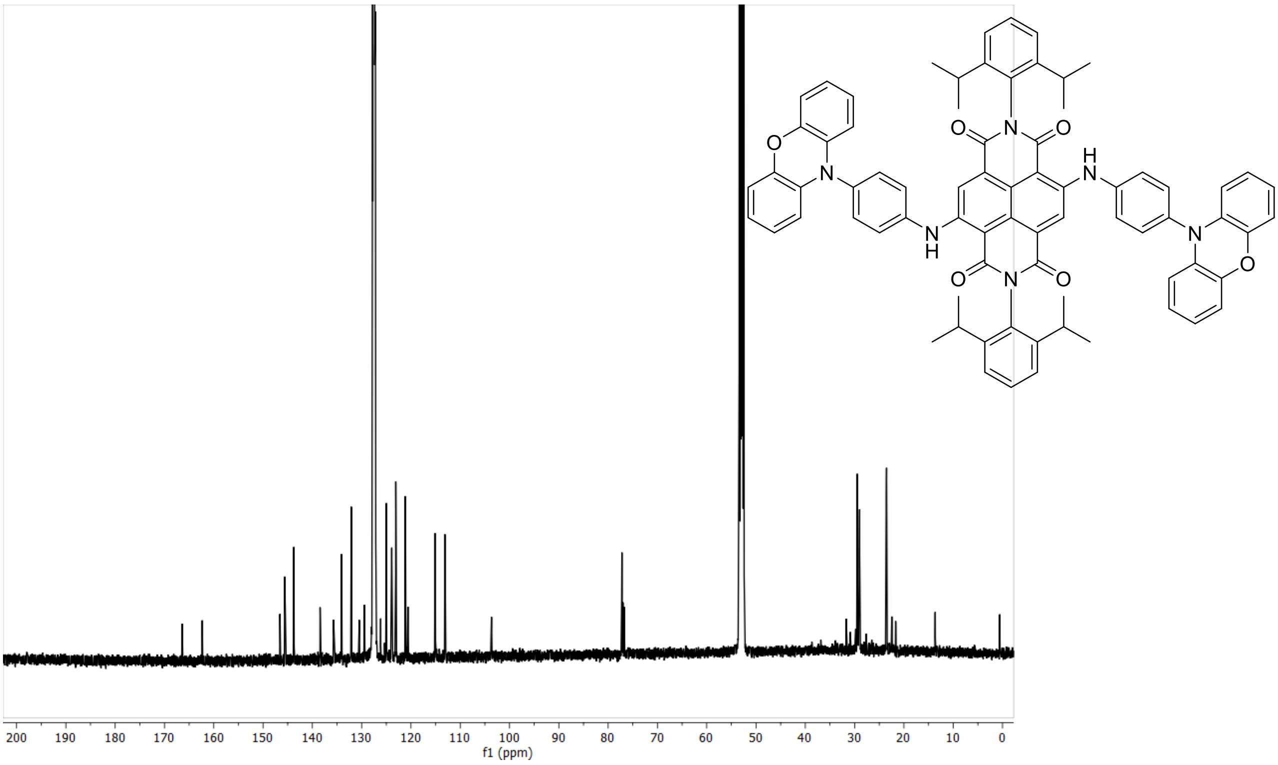

Figure S12. ^{13}C NMR Spectrum of compound **4**.

Compound **5**

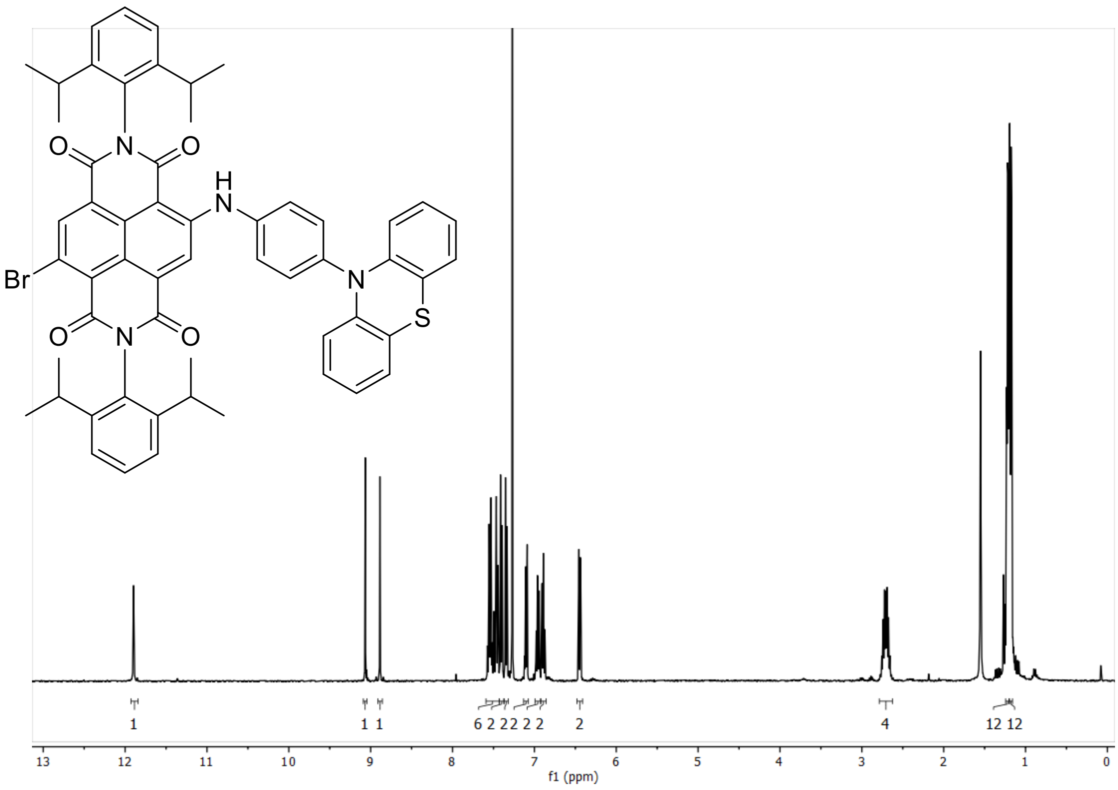

Figure S13. ^{1}H NMR Spectrum of compound **5**.

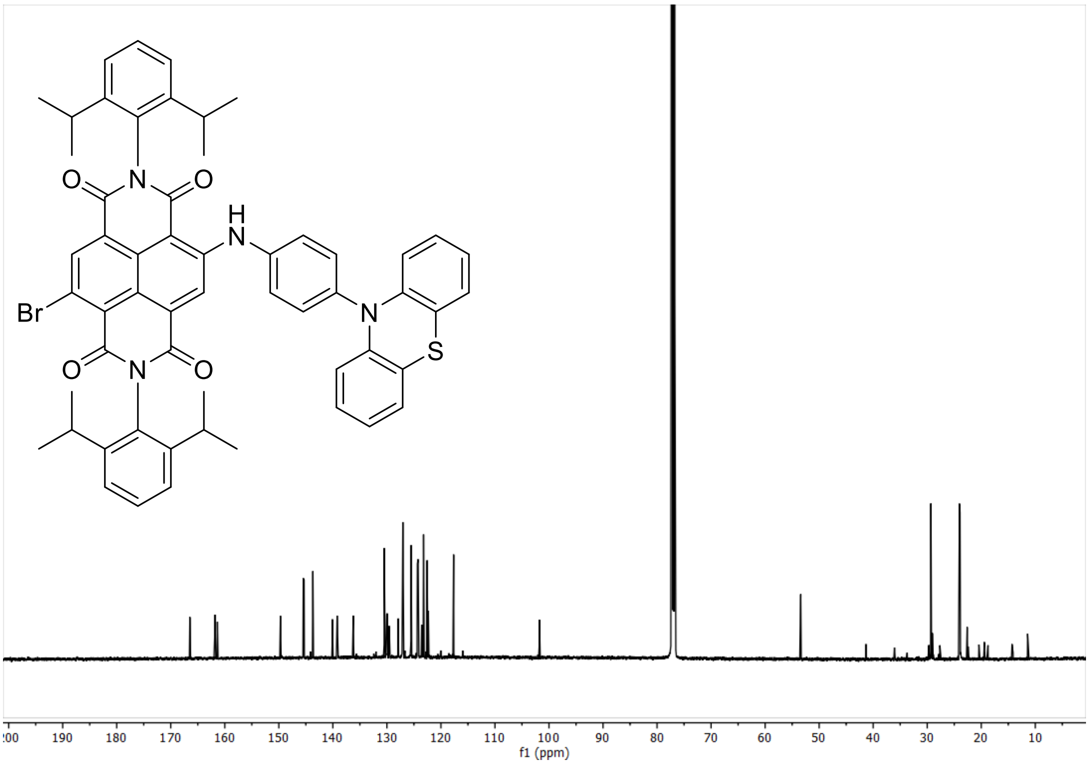

Figure S14. ^{13}C NMR Spectrum of compound **5**.

Compound **6**

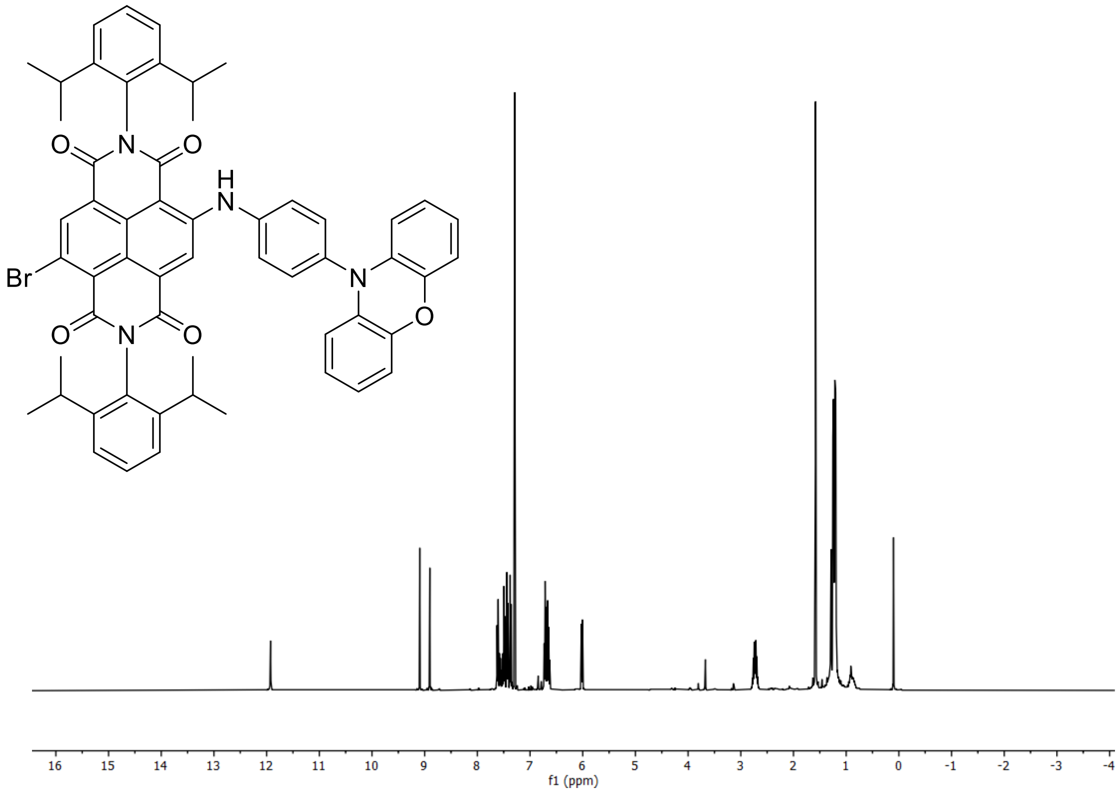

Figure S15. ^{1}H NMR Spectrum of compound **6**.

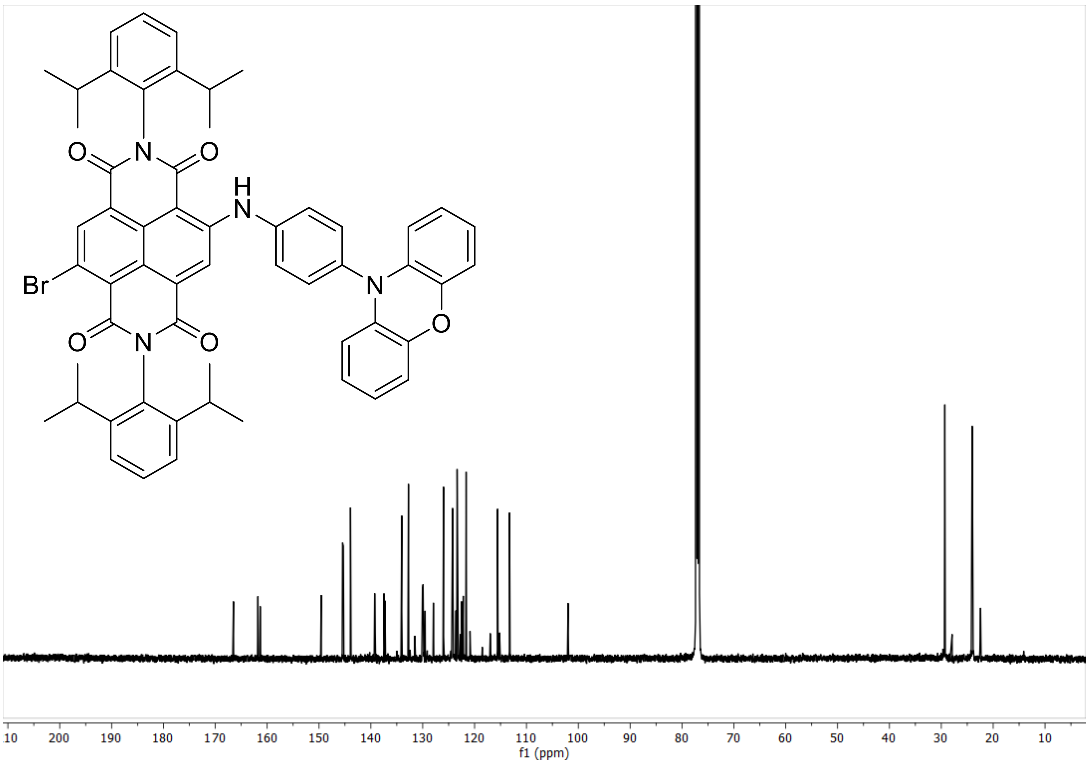

Figure S16. ^{13}C NMR Spectrum of compound **6**.

Compound **7**

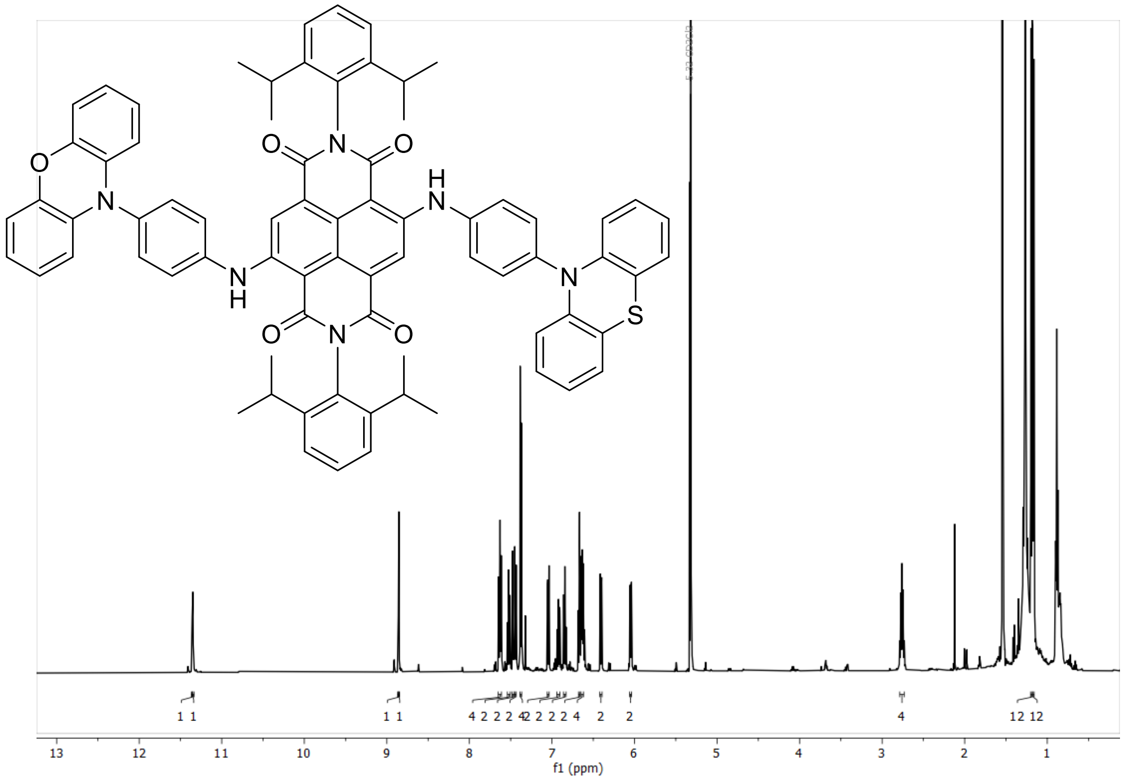

Figure S17. ^{1}H NMR Spectrum of compound **7**.

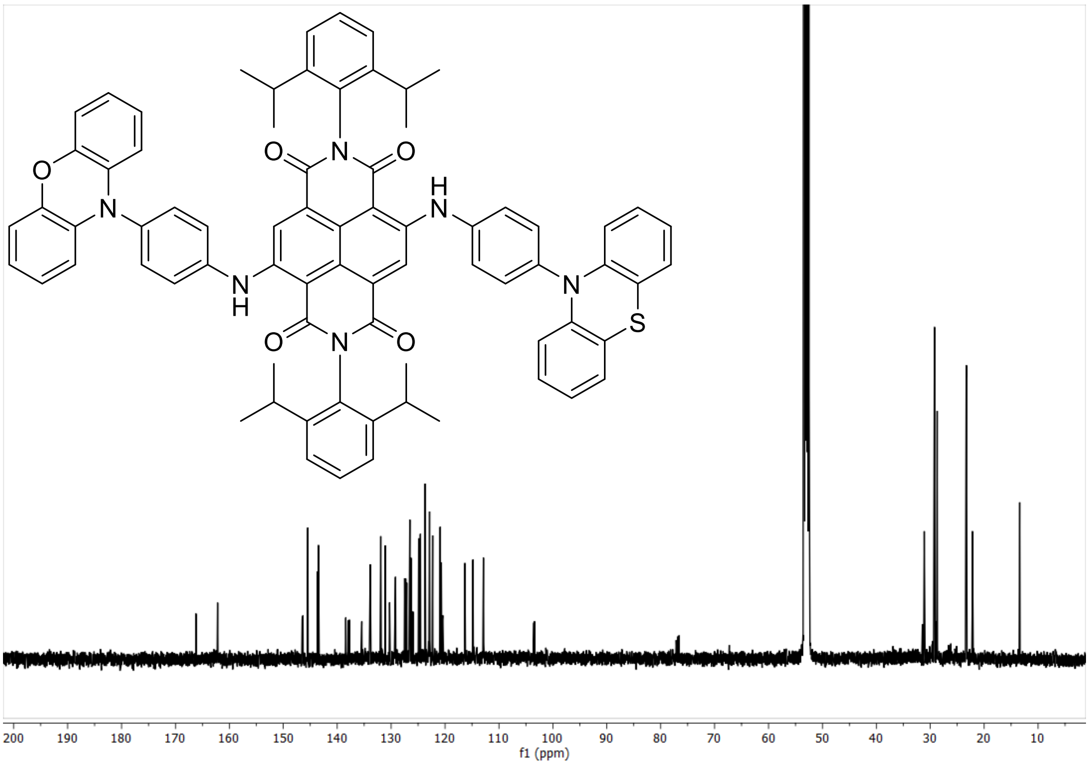

Figure S18. ^{13}C NMR Spectrum of compound **7**.

Compound **8**

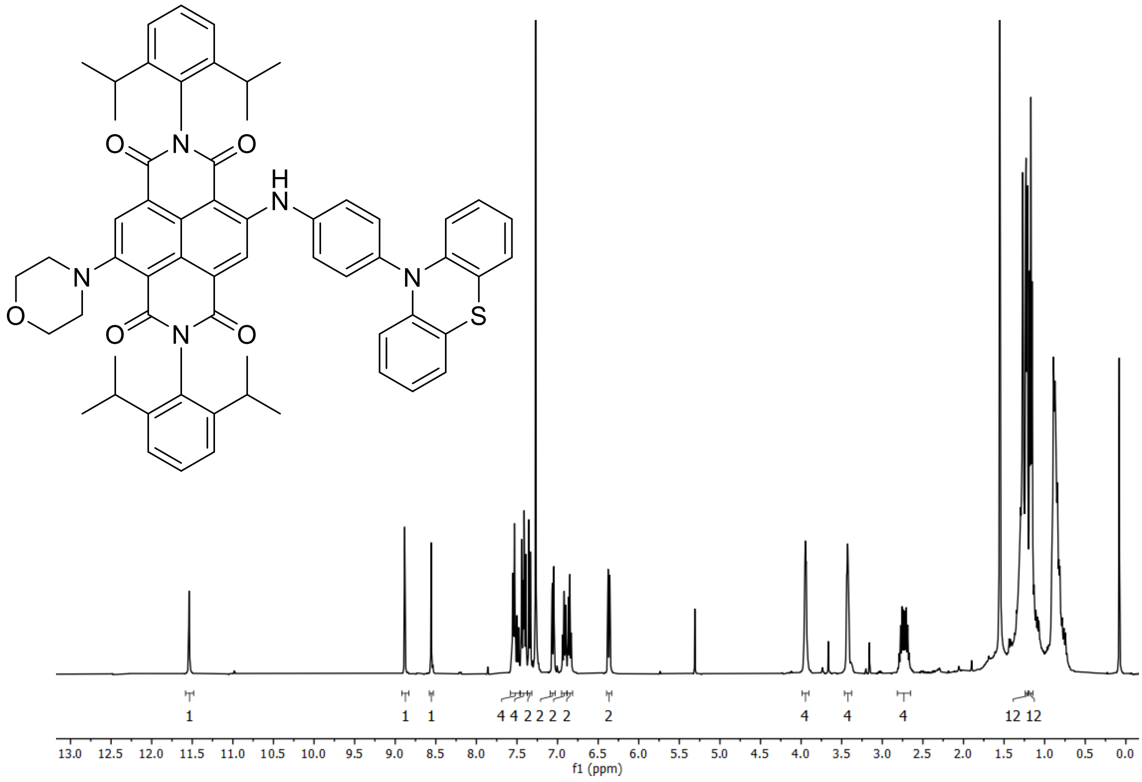

Figure S19. ^{1}H NMR Spectrum of compound **8**.

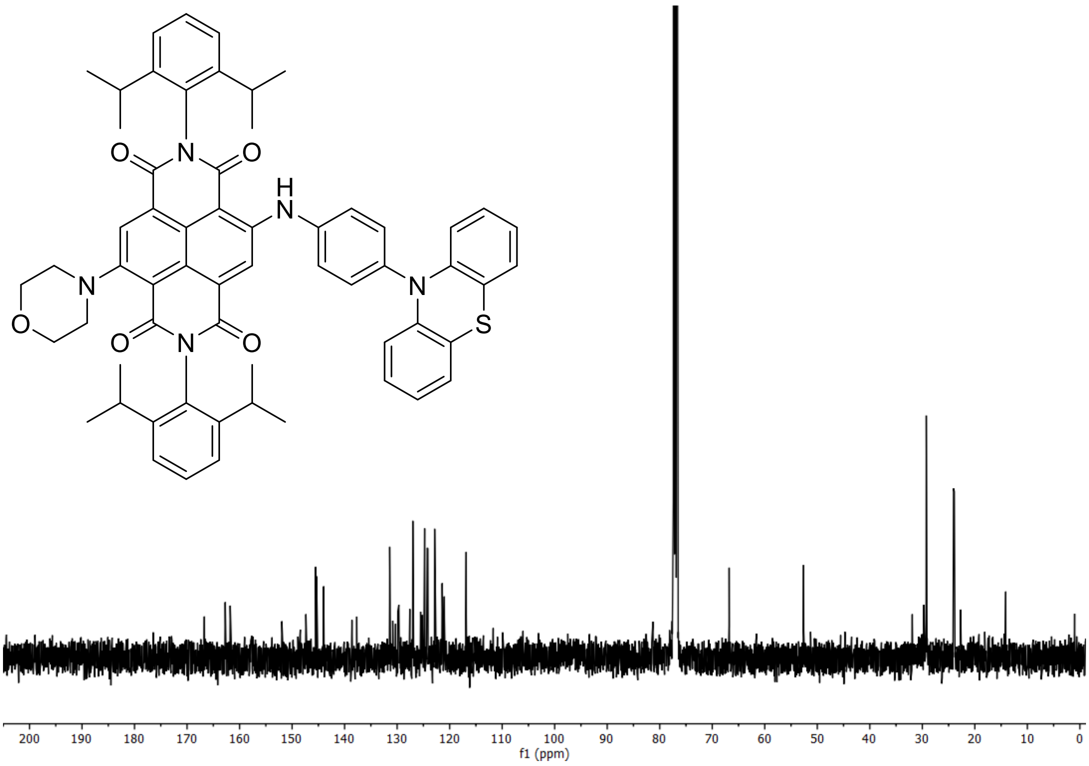

Figure S20. ^{13}C NMR Spectrum of compound **8**.

Compound **9**

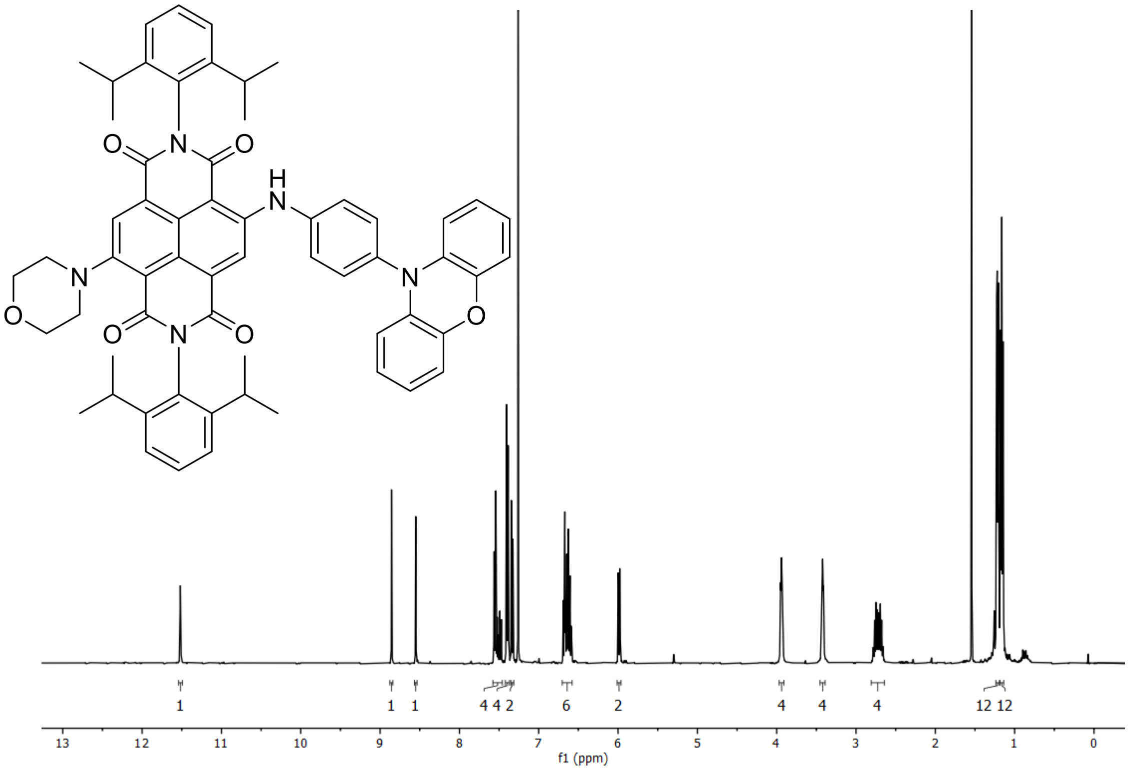

Figure S21. ^{1}H NMR Spectrum of compound **9**.

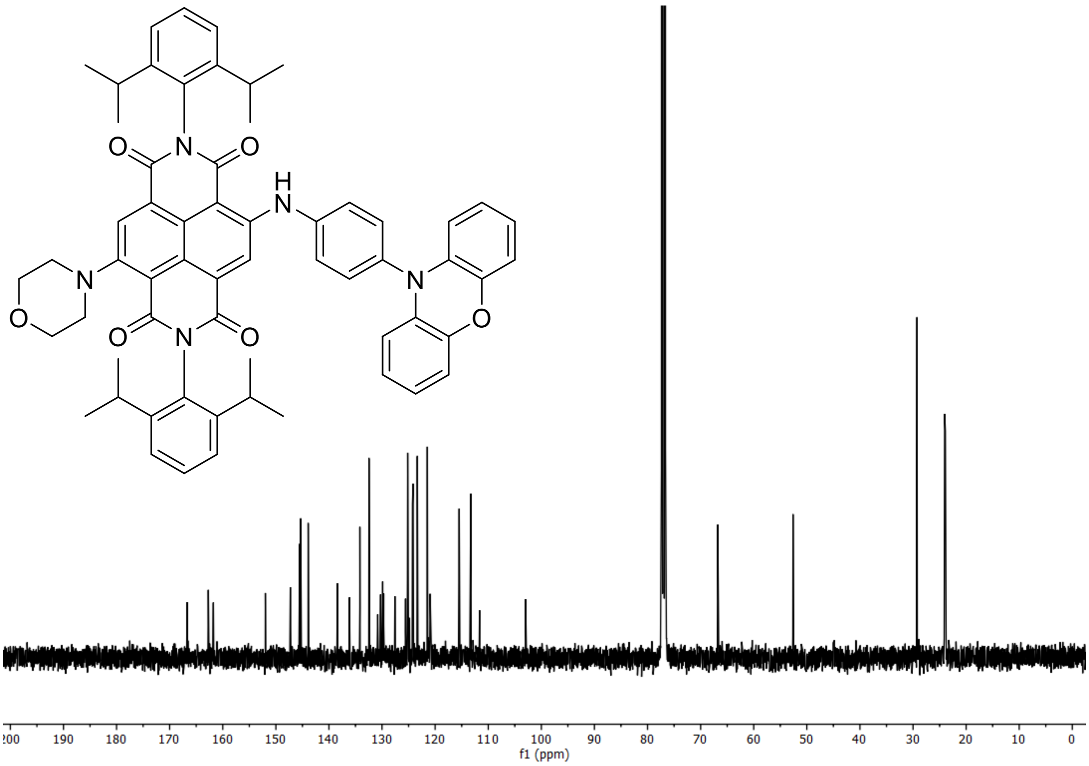

Figure S22. ^{13}C NMR Spectrum of compound **9**.

Additional Details for X-ray Crystallography

Single crystal diffraction data were collected at 120(2) K for **1, 5** and **9** on an Oxford Diffraction SuperNova CCD area detector diffractometer and for **2** on a Bruker GV1000 spectrometer using mirror monochromated Cu Kα radiation. Using Olex2,[S4] the structure was solved with the ShelXT[S5] structure solution program using Intrinsic Phasing and refined with the ShelXL[S6] refinement package using Least Squares minimisation. For specific details of modelling of disorder see CIFs for each structure.

Crystal Data for **1** $C_{66}H_{84}Cl_6N_4O_{12}S$ (*M* =1370.13 g/mol): orthorhombic, space group $P2_12_12_1$ (no. 19), *a* = 13.9099(6) Å, *b* = 16.2937(9) Å, *c* = 25.4691(10) Å, *V* = 5772.4(5) Å^3, *Z* = 4, *T* = 120(2) K, μ(CuKα) = 3.655 mm^{-1}, *Dcalc* = 1.577 g/cm^3, GooF = 1.087, 31326 reflections measured (6.44° ≤ 2Θ ≤ 147.646°), 11431 unique (R_{int} = 0.0961) which were used in all calculations. The final R_1 was 0.1124 (I > 2σ(I)) and wR_2 was 0.3515 (all data).

Crystal Data for **2** $C_{69}H_{99}Cl_3N_4O_{17}$ (*M* =1362.87 g/mol): orthorhombic, space group $P2_12_12_1$ (no. 19), *a* = 13.7486(5) Å, *b* = 15.9651(9) Å, *c* = 25.3392(12) Å, *V* = 5561.9(5) Å^3, *Z* = 4, *T* = 120(2) K, μ(CuKα) = 2.219 mm^{-1},*Dcalc* = 1.628 g/cm^3, GooF = 1.001, 28533 reflections measured (6.544° ≤ 2Θ ≤ 147.344°), 10919 unique (R_{int} = 0.0886) which were used in all calculations. The final R_1 was 0.0959 (I > 2σ(I)) and wR_2 was 0.2994 (all data).

Crystal Data for **5** $C_{56}H_{49}BrN_4O_4S$ (*M* = 953.95 g/mol): triclinic, space group P-1 (no. 2), *a* = 11.8382(7) Å, *b* = 14.9706(9) Å, *c* = 17.8355(11) Å, α = 98.319(5) °, *β* = 98.236(5) °, χ = 101.987(5) °, *V* = 3010.0(3) Å^3, *Z* = 2, *T* = 120(2) K, μ(CuKα) = 1.578 mm^{-1}, *Dcalc* = 1.053 g/cm^3, GooF = 1.043, 11788 reflections measured (7.760° ≤ 2Θ ≤ 147.47°), 7408 unique (R_{int} = 0.0689) which were used in all calculations. The final R_1 was 0.094 (I > 2σ(I)) and wR_2 was 0.2993 (all data).

Crystal Data for **9** $C_{252}H_{242}Cl_{36}N_{20}O_{25}$ (*M* =5226.84 g/mol): monoclinic, space group C2/c (no. 15), *a* = 41.9250(10) Å, *b* = 8.9649(4) Å, *c* = 33.8452(7) Å, *β* = 105.265(2)°, *V* = 12272.0(7) Å^3, *Z* = 2, *T* = 120(2) K, μ(CuKα) = 4.213 mm^{-1}, *Dcalc* = 1.414 g/cm^3, GooF = 1.026, 31610 reflections measured (7.804° ≤ 2Θ ≤ 147.512°), 12090 unique (R_{int} = 0.0246, R_{sigma} = 0.0231) which were used in all calculations. The final R_1 was 0.0527 (I > 2σ(I)) and wR_2 was 0.1373 (all data).

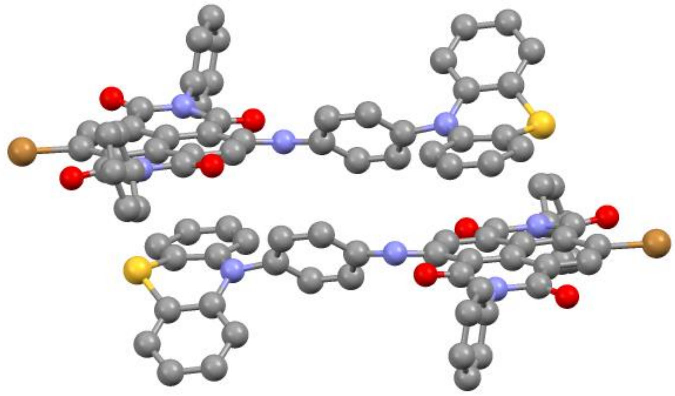

Figure S23. π-π stacking interactions between two molecules of **5** in the solid state. One ring of the phenothiazine group interacts with the NDI of an adjacent molecule. Using two such interactions, pairs of molecules are observed. Hydrogen atoms and the isopropyl groups are omitted for clarity. C – grey; O -red; N -blue; S – yellow; Br - brown.

Electrochemical and Optical Investigations

UV/visible absorption spectra were recorded on Perkin-Elmer Lambda 25 spectrometer. Cyclic voltammetric and coulometric studies were conducted using an Autolab PGSTAT20 potentiostat. DCM was dried *via* distillation under nitrogen over calcium hydride. [$^nBu_4N$][BF_4] was prepared *via* literature methods. Standard cyclic voltammetry was carried out under an atmosphere of argon using a three-electrode arrangement in a single compartment cell. Electrodes used the cell were as follows; A glassy carbon working electrode, a Pt wire secondary electrode and a saturated calomel reference electrode, chemically isolated from the test solution *via* a bridge tube containing electrolyte solution and fitted with a porous vycor frit. The solutions were 10^{-3} M in molecule of interest and 0.4 M in [Bu_4N][BF_4] as supporting electrolyte. Redox potentials are quoted versus the ferrocenium-ferrocene couple used as an internal reference. Compensation for internal resistance was not applied.

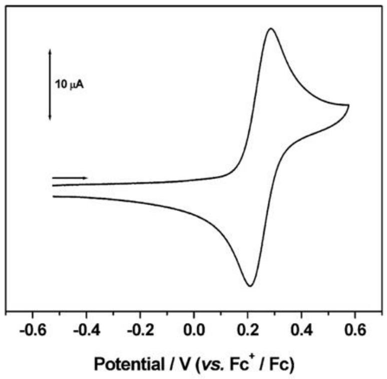

Figure S24. Cyclic voltammogram of POZ-NH_2 in CH_2Cl_2 with 0.4M[$^nBu_4$][BF_4] supporting electrolyte, at a scan rate of 100 mVs^{-1}.

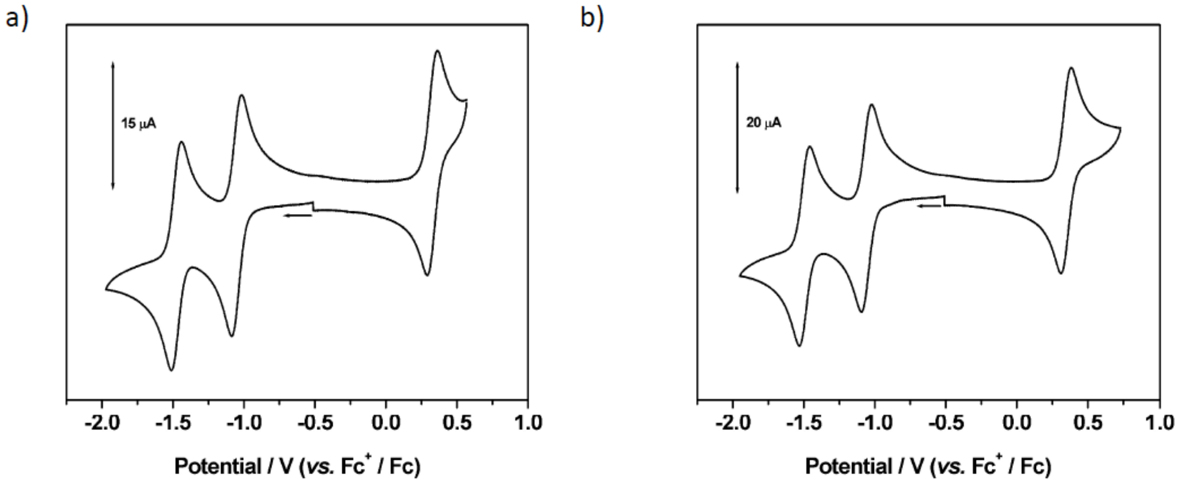

Figure S25. Cyclic voltammograms of a) **1** and b) **2** in CH_2Cl_2 with 0.4M[$^nBu_4$][BF_4] supporting electrolyte, at a scan rate of 100 mVs^{-1}.

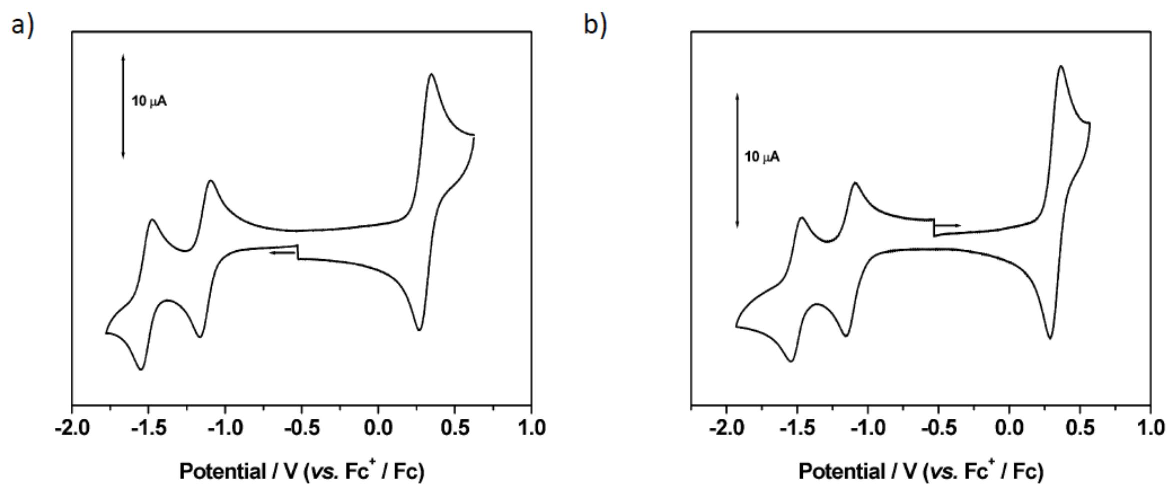

Figure S26. Cyclic voltammograms of a) **3**; b) **4** in CH_2Cl_2 with 0.4M[$^nBu_4$][BF_4] supporting electrolyte, at a scan rate of 100 mVs^{-1}.

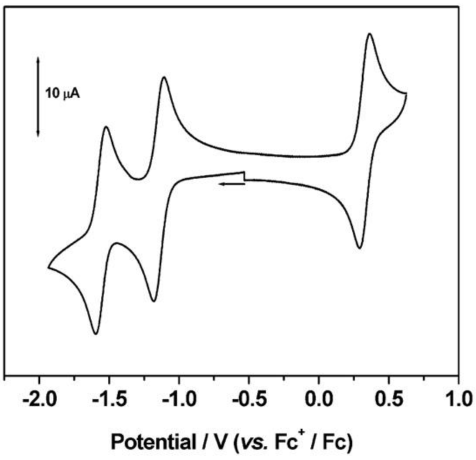

Figure S27. Cyclic voltammograms of **9** in CH_2Cl_2 with 0.4M [$^nBu_4$][BF_4] supporting electrolyte, at a scan rate of 100 mVs^{-1}.

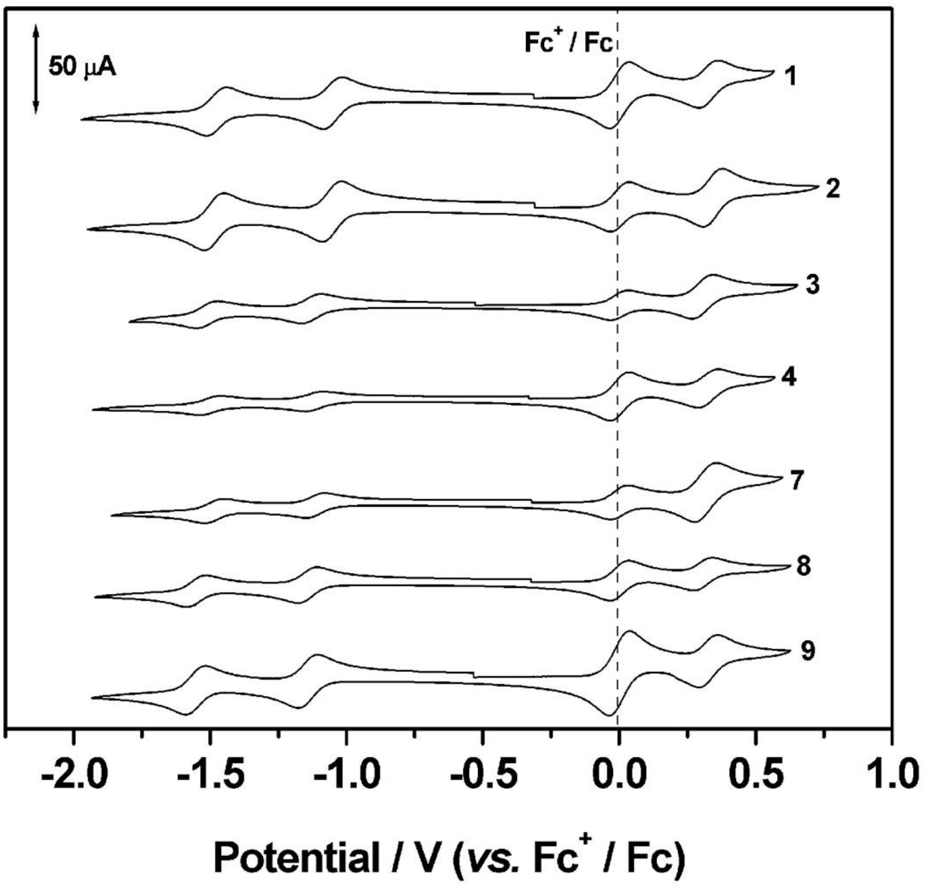

Figure S28. Cyclic voltammograms of compounds **1**-**9** combined with ferrocene in CH_2Cl_2 with 0.4M [$^nBu_4$][BF_4] supporting electrolyte, at a scan rate of 100 mVs^{-1}. Ferrocene is used to calibrate the potentials recorded and its redox potentials is highlighted with a dotted line.

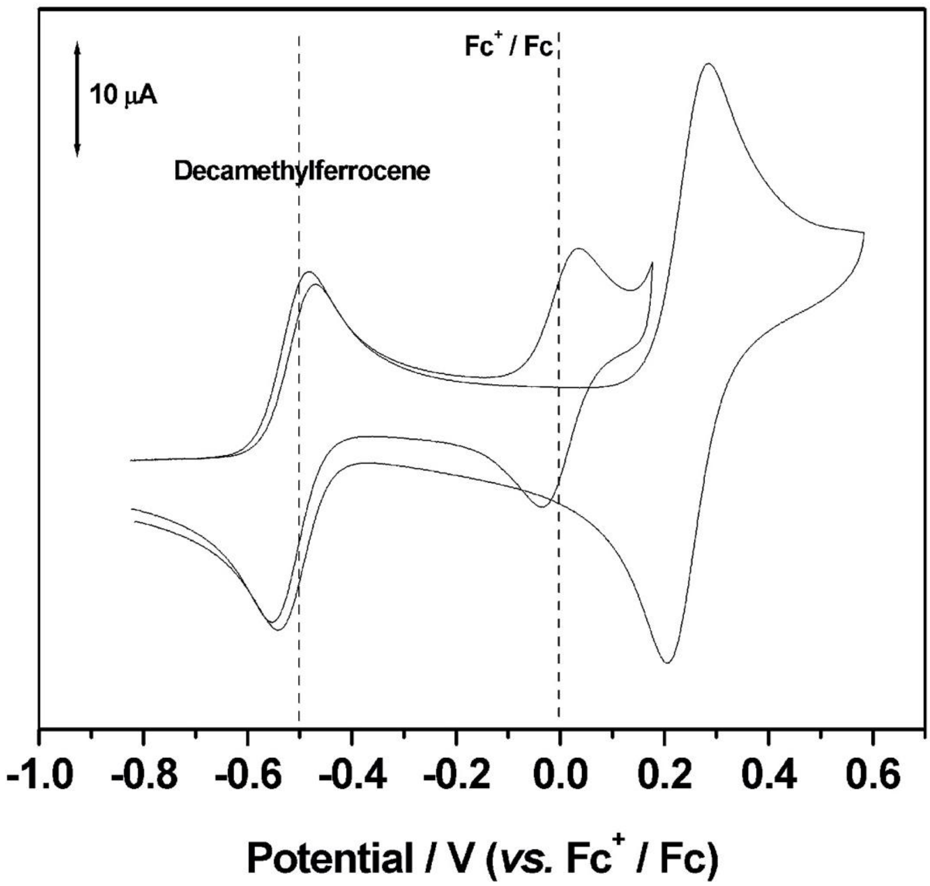

Figure S29. Cyclic voltammograms of a solution containing POZ-NH_2, ferrocene and decamethylferrocene in CH_2Cl_2 with 0.4M [$^{n}Bu_4$][BF_4] supporting electrolyte, at a scan rate of 100 mVs^{-1}. Ferrocene and decamethylferrocene are used to calibrate the potentials recorded and their redox potentials are highlighted with dotted lines.

Optical Spectroscopy and Spectroelectrochemistry

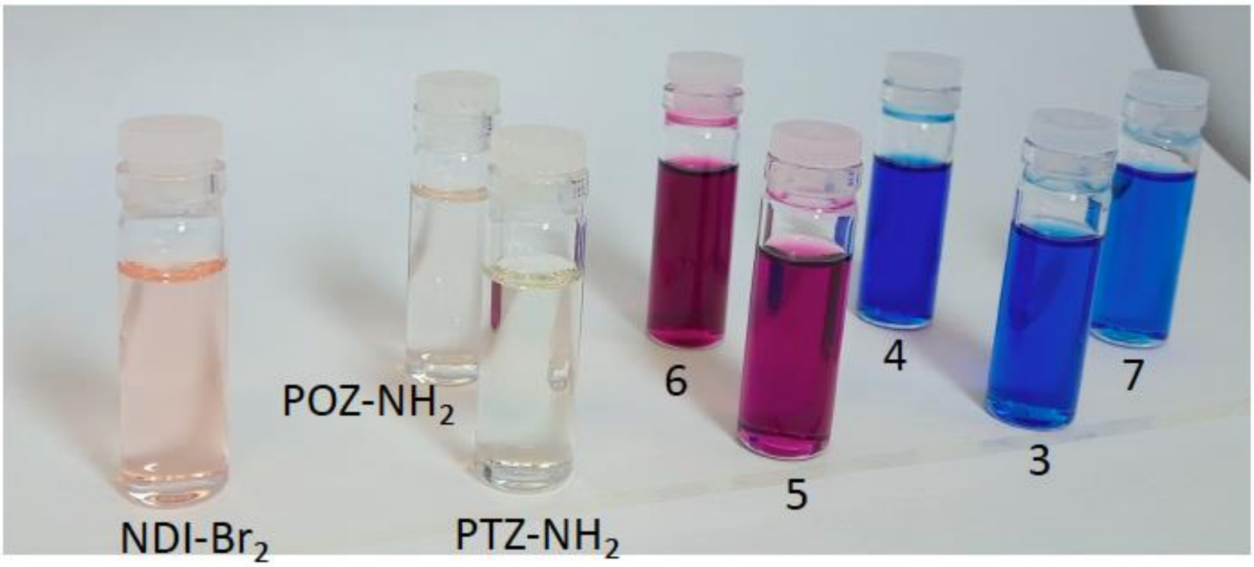

Figure S30. Photographs of CH_2Cl_2 solutions of the various compounds.

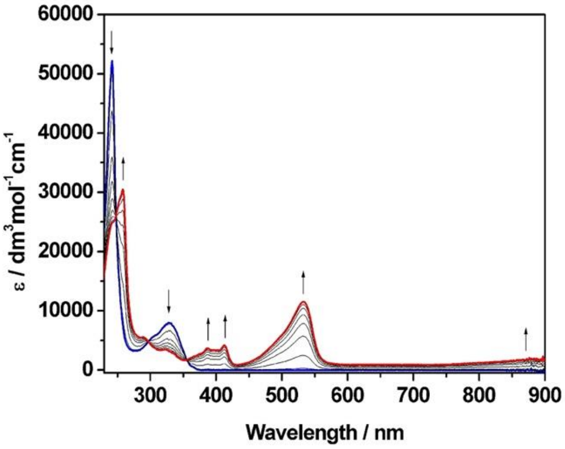

Figure S31. UV/vis absorption spectra showing the inter-conversion of POZ-NH$_2$ (blue) to [POZ-NH$_2$]$^{1+}$ (red) recorded in CH_2Cl_2 containing [Bu$_4$N][BF$_4$] (0.4 M) using spectroelectrochemical methods at 243 K. Arrows show the progress of the oxidation.

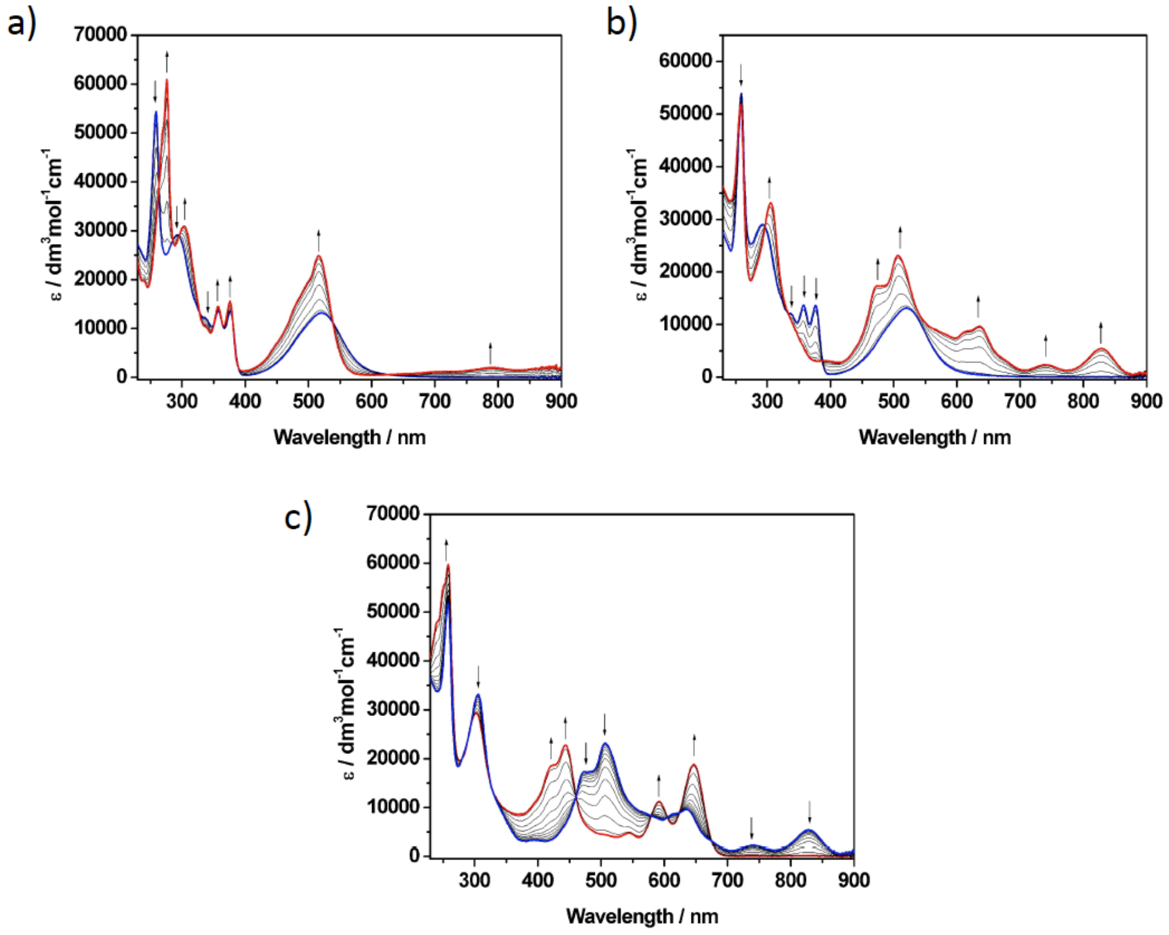

Figure S32. UV/vis absorption spectra recorded in CH_2Cl_2 containing $[Bu_4N][BF_4]$ (0.4 M) using spectroelectrochemical methods for **1** at 243 K showing a) the inter-conversion of **1** (blue) to $\mathbf{1}^{1+}$ (red); b) the inter-conversion of **1** (blue) to $\mathbf{1}^{1-}$ (red) and c) inter-conversion of $\mathbf{1}^{1-}$ (blue) to $\mathbf{1}^{2-}$ (red). Arrows show the progress of a) the oxidation or b/c) the reduction.

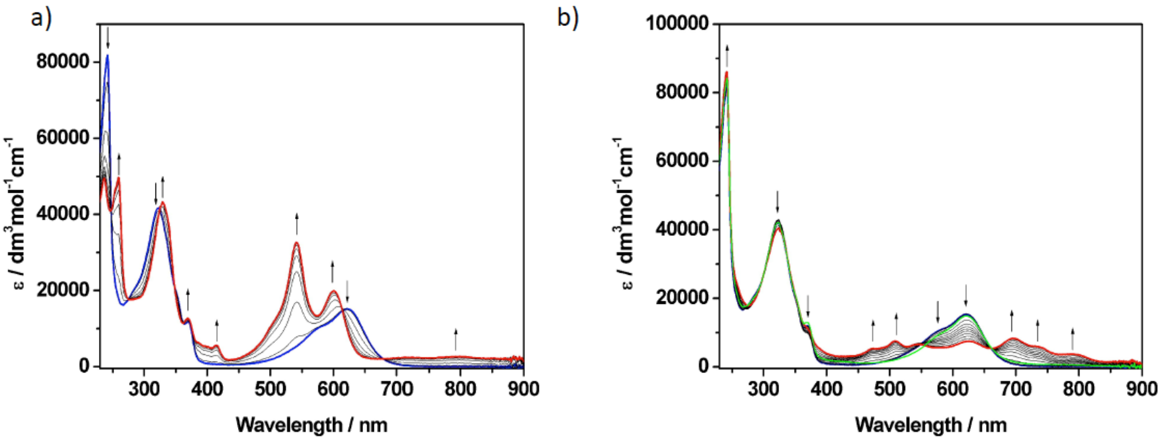

Figure S33. UV/vis absorption spectra recorded in CH_2Cl_2 containing $[Bu_4N][BF_4]$ (0.4 M) using spectroelectrochemical methods for **4** at 243 K showing a) the inter-conversion of **4** (blue) to $\mathbf{4}^{2+}$ (red); b) the

inter-conversion of **4** (blue) to $\mathbf{4}^{1-}$ (red) to the regenerated species (green). Arrows show the progress of a) the oxidation or b) the reduction.

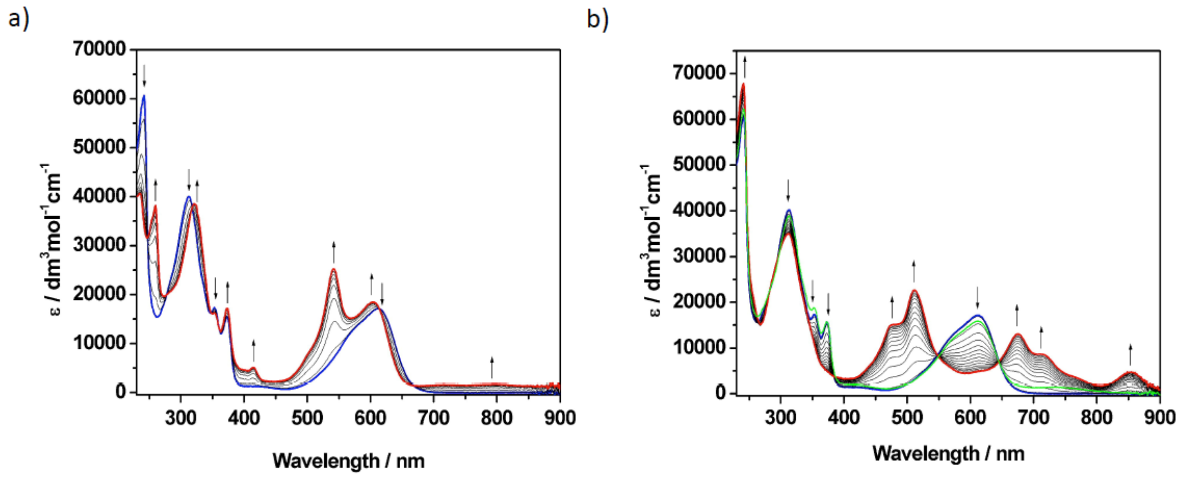

Figure S34. UV/vis absorption spectra recorded in CH_2Cl_2 containing $[Bu_4N][BF_4]$ (0.4 M) using spectroelectrochemical methods for **9** at 243 K showing a) the inter-conversion of **9** (blue) to $\mathbf{9}^{1+}$ (red); b) the inter-conversion of **9** (blue) to $\mathbf{9}^{1-}$ (red) to the regenerated species (green). Arrows show the progress of a) the oxidation and b) the reduction.

Table S1. Spectroelectrochemical data for selected compounds in their neutral, cationic, monoanionic and dianionic states.

**Compound**	λ_{abs} / *nm* (ε /10^3 mol^{-1}dm^{3}cm^{-1})
POZ-NH_2	242 (50.5), 330 (7.9)
[POZ-NH_2]$^+$	243 (253.2), 258 (30.5), 291 (5.6), 324 (3.5), 387 (3.7), 412 (4.2), 532 (11.6)
**1**	259 (54.3), 293 (29.3), 340 (11.9), 357 (13.7), 376 (13.6), 520 (13.2)
**1**$^+$	239 (19.9), 276 (61.2), 304 (31.0), 357 (14.6), 376 (15.7), 516 (25.1), 790 (2.0), 881 (2.3)
**1**$^-$	258 (52.0), 305 (33.3), 471 (17.4), 507 (23.4) 613 (8.8), 637 (9.9), 740 (2.5), 829 (5.7)
**1**$^{2-}$	240 (48.1), 250 (55.4), 258 (59.8), 303 (29.4), 422 (18.8), 443 (22.8), 544 (50.0), 590 (11.3), 646 (19.0)
**2**	241 (58.3), 292 (33.8), 337 (17.6), 357 (17.6), 376 (15.9), 517 (14.8)
**2**$^+$	237 (36.1), 260 (41.7), 305 (34.0), 357 (16.6), 375 (19.0), 515 (24.7), 537 (27.3), 784 (2.3)
**2**$^-$	240 (67.6), 303 (35.5), 475 (18.3), 506 (23.7), 612 (9.4), 635 (10.7), 741 (2.9), 827 (5.6)
**3**	258 (65.3), 325 (43.9), 371 (11.7), 574 (11.0), 621 (18.3)
**3**$^{2+}$	276 (70.1), 330 (46.9), 339 (43.7), 369 (13.6), 517 (15.5), 563 (12.1), 606 (20.2), 786 (2.6), 879 (2.7)
**3**$^-$	258 (75.5), 325 (50.1), 472 (11.2), 509 (17.0), 543 (12.3), 627 (4.8), 695 (16.2), 736 (10.2), 795 (4.8), 873 (3.2)
**4**	242 (81.6), 323 (42.0), 372 (12.1), 578 (10.4), 623 (15.4)
**4**$^{2+}$	235 (49.6), 260 (49.9), 330 (43.5), 369 (12.7), 415 (5.7), 541 (33.0), 601 (20.2), 799 (2.8)
**4**$^-$	241 (86.1), 323 (40.4), 470 (5.5), 508 (7.7), 546 (6.9), 626 (7.7), 695 (8.5), 737 (6.0), 794 (3.9)
**7**	242 (64.3), 256 (30.2), 324 (33.6), 371 (8.8), 579 (7.6), 624 (11.6)
**7**$^{2+}$	237 (41.2), 260 (43.1), 276 (30.1), 329 (33.5), 370 (9.9), 415 (4.1), 541 (20.9), 603 (14.5), 793 (2.1)
**7**$^-$	242 (65.9), 258 (31.7), 325 (33.8), 472 (6.4), 509 (10.0), 542 (7.5), 628 (3.0), 696 (9.4), 737 (5.9), 795 (2.9), 869 (1.8)
**7**$^{2-}$	241 (72.5), 256 (36.1), 318 (32.0), 436 (7.7), 460 (8.8), 560 (3.3), 609 (7.7), 668 (13.6)
**8**	258 (38.1), 314 (27.1), 354 (10.0), 373 (10.4), 612 (11.8)
**8**$^+$	276 (43.3), 319 (26.0), 353 (10.8), 373 (10.8), 518 (9.1), 606 (11.8), 790 (1.0)
**8**$^-$	258 (39.1), 314 (24.9), 475 (11.3), 512 (17.6), 674 (9.1), 716 (5.6), 853 (3.3)
**8**$^{2-}$	239 (38.1), 258 (41.9), 279 (18.7), 311 (20.0), 412 (12.2), 435 (14.1), 506 (3.4), 552 (3.0), 600 (8.6), 657 (15.5)
**9**	242 (60.1), 313 (39.9), 353 (17.2), 373 (15.3), 614 (16.9)
**9**$^+$	235 (40.2), 260 (37.9), 322 (38.3), 354 (16.1), 373 (17.1), 415 (5.2), 542 (25.1), 605 (18.5), 795 (1.6)
**9**$^-$	241 (67.2), 311 (34.8), 474 (14.9), 512 (22.7), 675 (13.0), 715 (8.7), 853 (4.8)

References

S1. Y. V. Suseela, M. Sasikumar, T. Govindaraju, *Tetrahedron Lett*., 2013, **54**, 6314–6318.

S2. S. Quinn, E.S. Davies, C.R. Pfeiffer, W. Lewis, J. McMaster, N. R. Champness, *ChemPlusChem*, **2017**, *82*, 489-492.

S3. E. A. Weiss, M. J. Ahrens, L. E. Sinks, A. V. Gusev, M. A. Ratner, M. R. Wasielewski, *J. Am. Chem. Soc*. **2004**, *126*, 5577−5584.

S4. O. V. Dolomanov, L. J. Bourhis, R.J. Gildea, J. A. K. Howard, H. Puschmann, *J. Appl. Cryst.* 2009, **42**, 339-341.

S5. G.M. Sheldrick, *Acta Cryst.*, 2015, **A71**, 3-8.

S6. G.M. Sheldrick, *Acta Cryst.*, 2015, **C71**, 3-8.
